# Inactivated cGAS‐STING Signaling Facilitates Endocrine Resistance by Forming a Positive Feedback Loop with AKT Kinase in ER+HER2– Breast Cancer

**DOI:** 10.1002/advs.202403592

**Published:** 2024-07-18

**Authors:** Kai‐Ming Zhang, De‐Chang Zhao, Ze‐Yu Li, Yan Wang, Jian‐Nan Liu, Tian Du, Ling Zhou, Yu‐Hong Chen, Qi‐Chao Yu, Qing‐Shan Chen, Rui‐Zhao Cai, Zi‐Xuan Zhao, Jia‐Lu Shan, Bing‐Xin Hu, Hai‐Liang Zhang, Gong‐Kan Feng, Xiao‐Feng Zhu, Jun Tang, Rong Deng

**Affiliations:** ^1^ State Key Laboratory of Oncology in South China Guangdong Provincial Clinical Research Center for Cancer Collaborative Innovation Center for Cancer Medicine Guangdong Key Laboratory of Nasopharyngeal Carcinoma Diagnosis and Therapy Sun Yat‐sen University Cancer Center Guangzhou 510060 China; ^2^ Department of Breast Oncology Sun Yat‐sen University Cancer Center Guangzhou 510060 China; ^3^ BGI Research Shenzhen 518083 China; ^4^ College of Life Sciences University of Chinese Academy of Sciences Beijing 100049 China; ^5^ Department of Oncology The Affiliated Yantai Yuhuangding Hospital of Qingdao University Yantai Shangdong 264000 China

**Keywords:** AKT kinase, cGAS‐STING pathway, endocrine‐resistant breast cancer, positive feedback loop

## Abstract

Endocrine‐resistant ER+HER2– breast cancer (BC) is particularly aggressive and leads to poor clinical outcomes. Effective therapeutic strategies against endocrine‐resistant BC remain elusive. Here, analysis of the RNA‐sequencing data from ER+HER2– BC patients receiving neoadjuvant endocrine therapy and spatial transcriptomics analysis both show the downregulation of innate immune signaling sensing cytosolic DNA, which primarily occurs in endocrine‐resistant BC cells, not immune cells. Indeed, compared with endocrine‐sensitive BC cells, the activity of sensing cytosolic DNA through the cGAS‐STING pathway is attenuated in endocrine‐resistant BC cells. Screening of kinase inhibitor library show that this effect is mainly mediated by hyperactivation of AKT1 kinase, which binds to kinase domain of TBK1, preventing the formation of a trimeric complex TBK1/STING/IRF3. Notably, inactivation of cGAS–STING signaling forms a positive feedback loop with hyperactivated AKT1 to promote endocrine resistance, which is physiologically important and clinically relevant in patients with ER+HER2– BC. Blocking the positive feedback loop using the combination of an AKT1 inhibitor with a STING agonist results in the engagement of innate and adaptive immune signaling and impairs the growth of endocrine‐resistant tumors in humanized mice models, providing a potential strategy for treating patients with endocrine‐resistant BC.

## Graphical abstract

In endocrine‐resistant breast, hyperactivated AKT1 blocks the downstream signal transduction of the cGAS‐STING pathway. In addition, inactivation of STING signal reduces its suppression on phosphorylated AKT1. The formation of positive feedback loop between inactivated STING signaling and hyperactivated AKT leads to further amplification of AKT1 phosphorylation and suppressed STING signaling in endocrine‐resistant breast cancer, promoting its endocrine resistance.

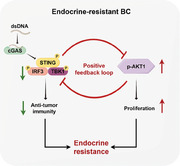

## 1 Introduction

Estrogen‐receptor‐positive (ER+) breast cancer accounts for ≈70% of all breast cancers.^[^ ^1^ ^]^ Although endocrine therapy has demonstrated a promising efficacy in reducing recurrence and improving the prognosis of patients with ER+ breast cancer, up to 40%–50% of patients eventually acquire resistance to endocrine therapy, which is the main cause of death in such patients.^[^ ^2^ ^]^ Many mechanisms of endocrine resistance in ER+ breast cancer have been reported.^[^ ^3^ ^]^ The drugs that have been approved for clinical application based on the mechanisms of endocrine resistance mainly include selective ER degraders, CDK4/6 inhibitors, HDAC inhibitors, PI3K/AKT/mTOR pathway inhibitors, tyrosine kinase inhibitors, etc.^[^ ^3^ ^]^ However, according to the reported results, the overall efficacy of these targeted drugs for endocrine‐resistant breast cancer is not satisfactory, and the objective response rate is ≈30%.^[^ ^4^ ^]^ In recent years, the efficacy of immunotherapy has been explored in patients with endocrine‐resistant breast cancer.^[^ ^5^, ^6^ ^]^ But the results of a clinical trial (NCT03051659) showed that the combination of PD‐1 antibody and chemotherapy had poor efficacy.^[^ ^6^ ^]^ So far, there are limited studies exploring the relationship between endocrine resistance and tumor immune microenvironment. Therefore, an in‐depth study of the characteristics and underlying molecular basis of tumor microenvironment in endocrine‐resistant breast cancer is crucial for developing effective treatment strategies.

The activation of innate immunity is important for the control of cancers.^[^ ^7^ ^]^ Recognition of cytosolic double‐stranded DNA (dsDNA) via the cGAS‐STING pathway is essential for endogenous innate immune sensing in cancer cells.^[^ ^8^ ^]^ The release of dsDNA fragments from the nucleus or mitochondria into the cytosol triggers the enzymatic function of cGAS to produce cyclic GMP‐AMP (cGAMP), which promotes the transport of STING from the endoplasmic reticulum (ER) to the Golgi apparatus to recruit TANK‐binding kinase 1 (TBK1) and IFN regulatory Factor 3 (IRF3).^[^ ^9^ ^]^ Then, the STING/TBK1/IRF3 trimer phosphorylates and dimerizes IRF3, and dimerized IRF3 is translocated into the nucleus to activate the type I interferon immune response,^[^ ^9^ ^]^ which not only inducing the intrinsic death of cancer cells, including apoptosis,^[^ ^10^ ^]^ ferroptosis,^[^ ^11^ ^]^ and PANoptosis,^[^ ^12^ ^]^ but also boosting antitumor immunity.^[^ ^13^ ^]^ A variety of malignant tumors adaptively silence cGAS‐STING signaling to promote tumor progression and immune escape.^[^ ^14^, ^15^, ^16^, ^17^ ^]^ Therefore, the cGAS‐STING pathway has been identified as a candidate drug target for pharmacological intervention against cancers,^[^ ^18^ ^]^ and STING agonists, such as ADU‐S100 and MK‐1454, have shown antitumor efficacy in clinical trials.^[^ ^19^ ^]^ However, the potential importance of the cGAS‐STING innate immune signal and its regulatory mechanism in endocrine‐resistant breast cancer remain largely unknown.

Here, we uncover that positive feedback loop of inactivated cGAS‐STING pathway and hyperactivated AKT1 is a crucial determinant of endocrine resistance in breast cancer. We demonstrate that hyperactivated AKT1 binds to TBK1 and disrupts downstream signaling from cGAS/STING to TBK1, meanwhile the inactivation of STING signaling could in turn promote hyperactivation of AKT1. The silencing of cGAS‐STING pathway promotes endocrine resistance by forming a positive feedback loop with hyperactivated AKT1. Importantly, the combination of STING agonists and AKT1 inhibitors effectively blocks this positive feedback loop to elicit potent activation of the cGAS‐STING pathway, resulting in the engagement of innate and adaptive immune signaling and suppressing the growth of endocrine‐resistant tumors. These findings support a treatment strategy that boosts potent antitumor immune responses and overcomes endocrine resistance in endocrine‐resistant breast cancer.

## 2 Results

### 2.1 Downregulation of Innate Immune Signaling Sensing Cytosolic DNA is Associated with Endocrine Resistance and Poor Prognosis in ER+HER2– BC Patients

**Figure 1:**
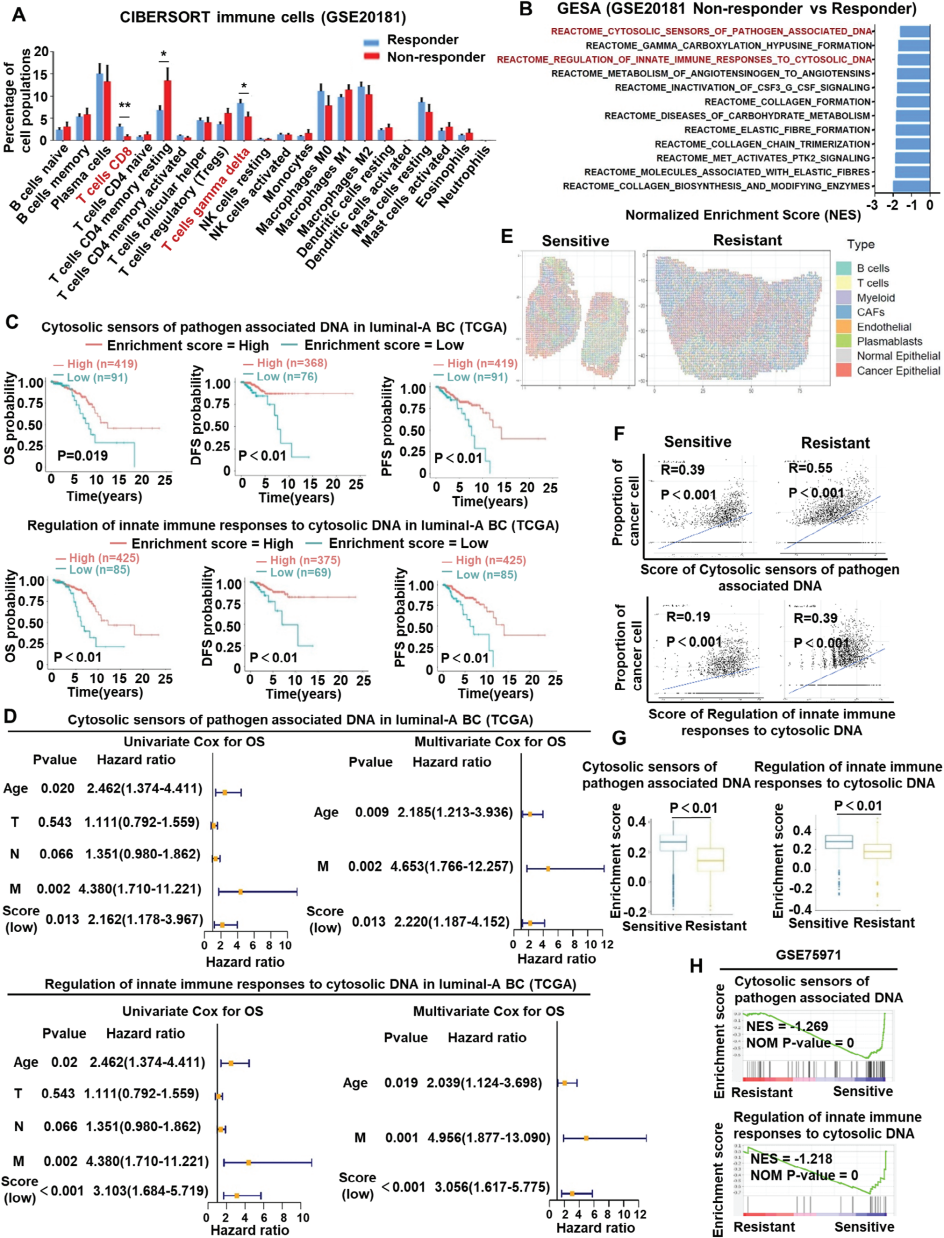
Innate immune signaling sensing cytosolic DNA were downregulated in endocrine‐resistant breast cancer cell. A) Tumor‐infiltrating immune cells based on CIBERSORT analysis of tumor‐infiltrating immune cells between responder and nonresponder for neoadjuvant endocrine therapy in GSE20181 data set. B) GSEA analysis of different REACTOME pathways between responder and nonresponder in GSE20181 data set. C) Kaplan–Meier analyses of overall survival (OS), disease free survival (DFS), and progression free survival (PFS) based on enrichment score of the innate immune signaling sensing cytoplasmic DNA for patients with Luminal‐A breast cancer. The data were retrieved from TCGA database. D) Univariate Cox regression analysis and multivariate Cox regression analysis regarding OS for patients with Luminal‐A breast cancer using the TCGA database. E) Visualization of the spatial distribution of cell types by spatial transcriptomics analysis. F) Pearson correlation analysis for the correlation between the abundance of tumor cell and the enrichment score of innate immune signal sensing cytosolic DNA. G) ssGSEA analysis of innate immune signal sensing cytosolic DNA in the tumor cells of endocrine‐resistant and endocrine‐sensitive breast cancer. H) Enrichment plot of cytosolic sensors of pathogen associated DNA pathway and regulation of innate immune responses to cytosolic DNA pathway based on GSEA analysis between endocrine‐sensitive and endocrine‐resistant cell lines in GSE75971 data set.

To investigate the features of the tumor microenvironment in endocrine‐resistant breast cancer, we first analyzed the RNA‐sequencing data (GSE20181) of ER+HER2– breast cancer patients treated with neoadjuvant endocrine therapy (letrozole) after 90 days, which could rule out other treatment interference. CIBERSORT analysis showed that the abundance of CD8^+^ T cells and γδ T cells was significantly lower in breast cancer tissues from nonresponders (*n* = 14) than in those from responders (**Figure** **1**A; and Figure S1A, Supporting Information). Furthermore, GSEA showed that the innate immune signaling sensing cytoplasmic DNA (Reactome cytosolic sensors of pathogen associated DNA, Reactome regulation of innate immune responses to cytosolic DNA) were significantly less enriched in nonresponders than responders (Figure 1B; and Figure S1B, Supporting Information), indicating that the innate immune signaling sensing cytoplasmic DNA might be involved in the antitumor immune response in endocrine‐resistant breast cancer. Next, we analyzed the clinical significance of innate immune signaling sensing cytoplasmic DNA in luminal‐A breast cancer patients of TCGA dataset. Kaplan–Meier survival analysis revealed that a low enrichment score of the innate immune signaling sensing cytoplasmic DNA was associated with poor overall survival (OS), disease‐free survival (DFS), and progression‐free survival (PFS) in patients with luminal‐A breast cancer (Figure 1C). Furthermore, multivariate Cox regression analysis showed that the enrichment score was also an independent prognostic factor for OS, DFS, and PFS in patients with luminal‐A breast cancer (Figure 1D; and Figure S1C,D, Supporting Information). It was reported that activation of innate immune signaling sensing cytoplasmic DNA in different types of cells could lead to different biological effects.^[^ ^20^, ^21^, ^22^ ^]^ To further investigate the specific cell types in which innate immune signaling sensing cytosolic DNA was downregulated in endocrine‐resistant breast cancer tissues, spatial transcriptomics analysis was performed on 4 primary breast cancer samples from endocrine‐sensitive (*n* = 2) and endocrine‐resistant (*n* = 2) patients. The spatial distribution of cell types showed that the abundance of tumor‐infiltrating T cells in endocrine‐resistant breast cancer tissues was significantly lower than that in endocrine‐sensitive breast cancer tissues (Figure 1E). Pearson correlation analysis showed that the abundance of cancer cells was significantly positively correlated with the enrichment score of innate immune signaling sensing cytosolic DNA (Figure 1F), while there was no positive correlation between the enrichment score and the abundance of other cell types, such as B cells, CAFs, T cells, myeloid cells, endothelial cells, and plasma blasts (Figure S1E–H, Supporting Information). ssGSEA showed that the enrichment score in endocrine‐resistant breast cancer cells was significantly lower than that in endocrine‐sensitive breast cancer cells (Figure 1G; and Figure S1I, Supporting Information). These results suggested that it might be cancer cells in which the innate immune signal sensing cytosolic DNA was downregulated. To further confirm this observation, we performed RNA‐sequencing analysis of an endocrine‐resistant breast cancer cell line, which was constructed by long‐term estrogen deprivation culture (GSE75971) (Figure S1J, Supporting Information). GSEA showed that innate immune signals sensing cytoplasmic DNA were significantly downregulated in endocrine‐resistant cell lines (Figure 1H). In addition, among the 330 genes downregulated in endocrine‐resistant cells, 54 immune‐regulated genes (IRGs) were identified, including 48 interferon signaling genes and 13 antigen presentation and processing genes (Figure S1K, Supporting Information). Among these IRGs, the type I interferon signaling‐associated genes IFNB1, IFIT1, and ISG15 were significantly downregulated in endocrine‐resistant cells (Figure S1L, Supporting Information), indicating that type I interferon signaling was suppressed in endocrine‐resistant cells. Collectively, these results from the clinical sample analysis suggest that compared with that in endocrine‐sensitive breast cancer, the innate immune signaling sensing cytosolic DNA is downregulated in endocrine‐resistant breast cancer cells, which is associated with the prognosis of ER+HER2– breast cancer patients.

### 2.2 cGAS‐STING Signaling is Inactive in Endocrine‐Resistant Breast Cancer Cells

**Figure 2:**
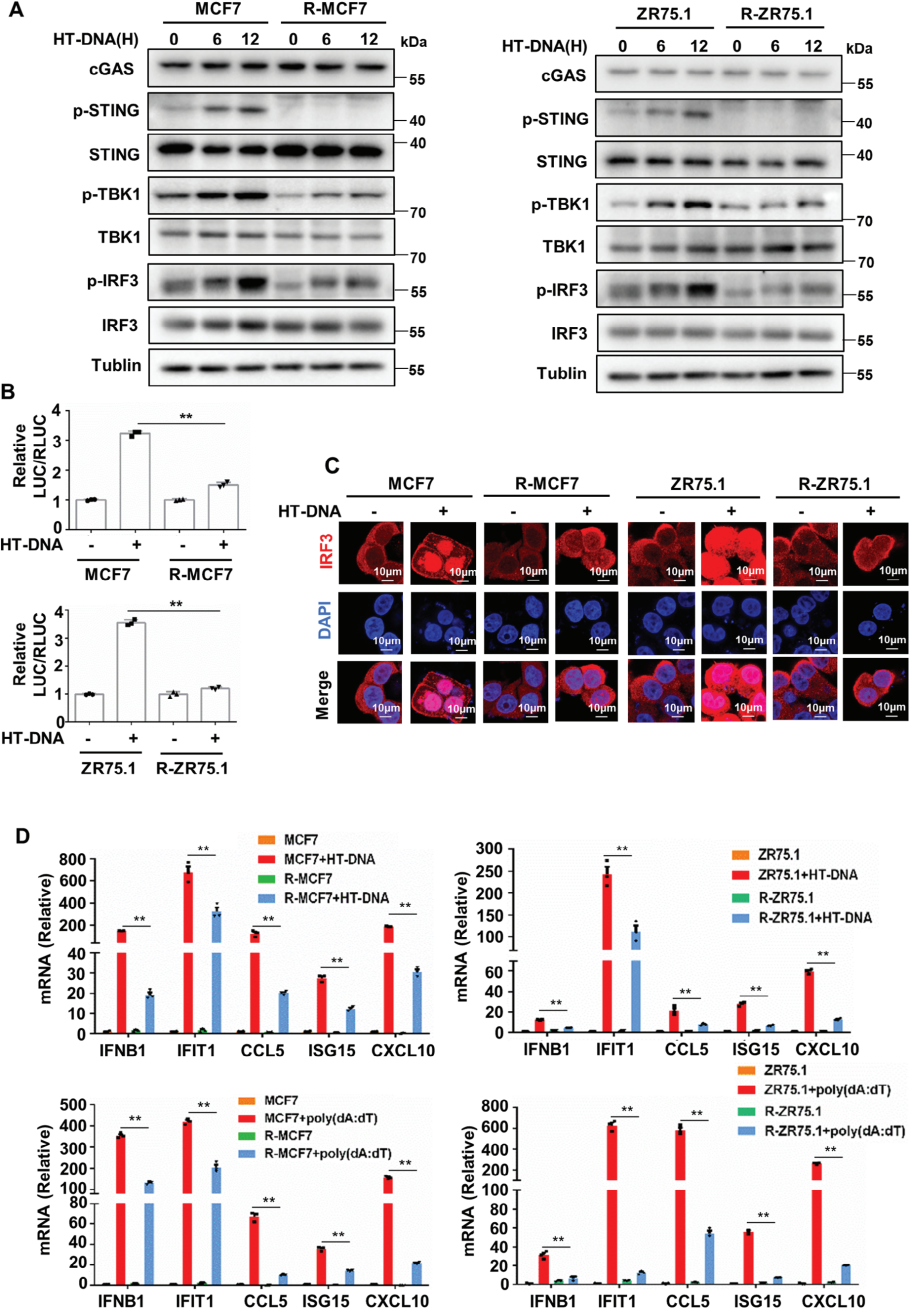
cGAS‐STING pathway is inactive in endocrine‐resistant breast cancer cells. A) MCF7/ZR75.1 cells and R‐MCF7/R‐ZR75.1 cells were treated with 2 µg mL^−1^ HT‐DNA for 6 or 12 h and harvested for western blot analysis of proteins in cGAS‐STING pathway. B) A luciferase‐reporter assay with an IRF3‐responsive ISRE promoter stimulated by cGAS‐STING pathway was used in MCF7/ZR75.1 cells and R‐MCF7/R‐ZR75.1 cells, after treated with 2 µg mL^−1^ HT‐DNA for 16 h, luciferase activity was detected (*n* = 3 biological independent samples). C) MCF7/ZR75.1 cells and R‐MCF7/R‐ZR75.1 cells were treated with 2 µg mL^−1^ HT‐DNA for 12 h and harvested for immunofluorescence detection of IRF3 (red). D) MCF7/ZR75.1 cells and R‐MCF7/R‐ZR75.1 cells were treated with 2 µg mL^−1^ HT‐DNA for 12 h or 200 ng mL^−1^ poly(dA:dT) for 24 h, then harvested for RT‐qPCR analysis of IFNB1 mRNA and ISGs mRNA (*n* = 3 biological independent samples). *p*‐values were calculated by unpaired two‐tailed Student's *t*‐test, **p <0.01. All data are representative of three independent experiments.

To further elucidate the role of innate immune signaling sensing cytosolic DNA in endocrine‐resistant breast cancer, we constructed the endocrine‐resistant breast cancer cell lines R‐MCF7 and R‐ZR75.1 by long‐term estrogen deprivation culture (Figure S2A, Supporting Information). Cell proliferation assays showed that endocrine‐resistant R‐MCF7/R‐ZR75.1 cells proliferated much faster than parental MCF7/ZR75.1 cells in estrogen‐deprived medium (Figure S2B, Supporting Information). In addition, we found that the colony formation ability of MCF7/ZR75.1 cells was significantly reduced with the decrease in estrogen in the medium, while the colony formation ability of R‐MCF7/R‐ZR75.1 cells was not significantly inhibited by the decrease in estrogen in the medium (Figure S2C, Supporting Information). The cGAS‐STING pathway is the major innate immune pathway that senses cytosolic DNA and activates interferon signaling.^[^ ^23^ ^]^ To determine whether the cGAS‐STING pathway is altered in endocrine‐resistant breast cancer cells, we initially transfected herring testis DNA (HT‐DNA) into MCF7/ZR75.1 cells and R‐MCF7/R‐ZR75.1 cells. Our results showed that HT‐DNA transfection in MCF7/ZR75.1 cells significantly increased the phosphorylation of TBK1 and its substrates. In contrast, the increased phosphorylation of these proteins, induced by HT‐DNA, was significantly inhibited in R‐MCF7/R‐ZR75.1 cells (**Figure** **2**A). IRF3 is the key transcription factor of the cGAS‐STING pathway.^[^ ^8^ ^]^ Consistent with the above results, a dual‐luciferase reporter assay showed that the increased transcriptional activity of IRF3, induced by HT‐DNA, in R‐MCF7/R‐ZR75.1 cells was significantly lower than that in MCF7/ZR75.1 cells (Figure 2B). Next, we examined the subcellular localization of IRF3. In MCF7/ZR75.1 cells, IRF3 was present in the cytoplasm, and most IRF3 was translocated into the nucleus after HT‐DNA treatment. In contrast, R‐MCF7/R‐ZR75.1 cells blunted the response to HT‐DNA treatment, resulting in significantly less IRF3 translocated into the nucleus (Figure 2C). Furthermore, we checked the dsDNA‐induced expression of the IRF3 target IFNB1 and various downstream cytokines. RT‐qPCR analysis showed that the mRNA expression of IFNB1, IFIT1, CCL5, ISG15, and CXCL10 was significantly increased in MCF7/ZR75.1 cells after transfection with HT‐DNA or poly(dA:dT). In contrast, the increased mRNA expression of these ISGs was significantly inhibited in R‐MCF7/R‐ZR75.1 cells (Figure 2D). Overall, our data indicate that endocrine‐resistant breast cancer cells are more insensitive to dsDNA‐induced activation of the cGAS‐STING pathway than parental cells.

### 2.3 Suppression of the cGAS‐STING Pathway in Endocrine‐Resistant Breast Cancer Mediates Immune Escape

**Figure 3:**
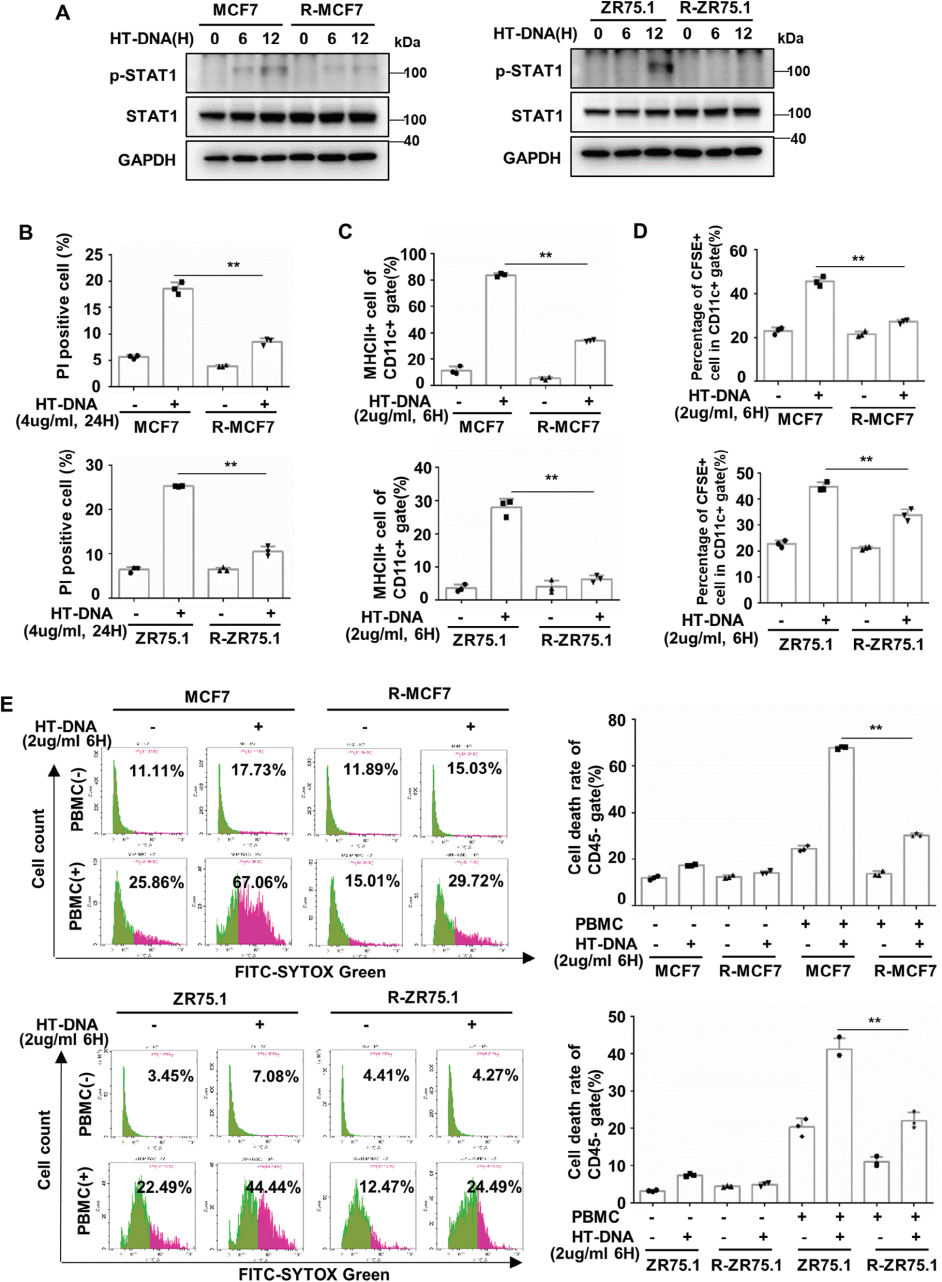
Inactivation of cGAS‐STING pathway in endocrine‐resistant breast cancer mediates immune escape. A) MCF7/ZR75.1 cells and R‐MCF7/R‐ZR75.1 cells were treated with 2 µg mL^−1^ HT‐DNA for 6 or 12 h and harvested for western blot analysis of phosphorylated STAT1. B) MCF7/ZR75.1 cells and R‐MCF7/R‐ZR75.1 cells were treated with 4 µg mL^−1^ HT‐DNA for 24 h and harvested for Annexin V‐FITC/PI staining assay (*n* = 3 biological independent samples). C) MCF7/ZR75.1 cells and R‐MCF7/R‐ZR75.1 cells were pretreated with 2 µg mL^−1^ HT‐DNA for 6 h, then cocultured with immature DC for 24 h and harvested for flow cytometry analysis of DC mature marker, MHC II (*n* = 3 biological independent samples). D) MCF7/ZR75.1 cells and R‐MCF7/R‐ZR75.1 cells were pretreated with 2 µg mL^−1^ HT‐DNA for 6 h, then dyed by CFSE and cocultured with immature DC for 16 h and harvested for flow cytometry analysis of DC that engulfing the cancer cells (*n* = 3 biological independent samples). E) MCF7/ZR75.1 cells and R‐MCF7/R‐ZR75.1 cells were pretreated with 2 µg mL^−1^ HT‐DNA for 6 h, then cocultured with PBMC (present with anti‐CD3, anti‐CD28, and IL‐2) for 72 h and harvested for flow cytometry analysis of cell death rate in cancer cells (*n* = 3 biological independent samples). *p*‐values were calculated by unpaired two‐tailed Student's *t*‐test, **p < 0.01. All data are representative of three independent experiments.

Cytosolic DNA activates the cGAS‐STING pathway to induce IFN‐JAK/STAT‐dependent cell death.^[^ ^24^ ^]^ First, we observed that the expression level of p‐STAT1 in MCF7/ZR75.1 cells was significantly higher than that in R‐MCF7/R‐ZR75.1 cells when tumor cells were transfected with HT‐DNA (**Figure** **3**A). In agreement, the cell death rate (PI staining positive) of R‐MCF7/R‐ZR75.1 cells was significantly less than that of MCF7/ZR75.1 cells when cells were treated with HT‐DNA (Figure 3B; and Figure S3B, Supporting Information). cGAS‐STING innate immune signaling also suppresses tumors by promoting antigen presentation and enhancing the cytotoxicity of immune cells.^[^ ^8^ ^]^ DCs are the main antigen‐presenting cells that link innate and adaptive immunity.^[^ ^25^ ^]^ Therefore, we determined the activation of DCs and the cytotoxicity of PBMCs when they were cocultured with HT‐DNA‐treated tumor cells. The results showed that HT‐DNA‐treated MCF7/ZR75.1 cells markedly increased the surface expression of the activation marker MHC‐II on DCs when the tumor cells were cocultured with DCs, while HT‐DNA‐treated R‐MCF7/R‐ZR75.1 cells failed to activate DCs (Figure 3C; and Figure S3A,D, Supporting Information). Furthermore, HT‐DNA‐treated MCF7/ZR75.1 cells significantly increased the percentage of DCs (CD11c+, CFSE+) that engulfed CFSE‐labeled tumor cells when the tumor cells were cocultured with DCs, while HT‐DNA‐treated R‐MCF7/R‐ZR75.1 cells slightly increased the percentage of DCs that engulfed CFSE‐labeled tumor cells (Figure 3D; and Figure S3C,F, Supporting Information). In addition, compared with that of MCF7/ZR75.1 cells, R‐MCF7/R‐ZR75.1 cells significantly reduced the percentage of dead tumor cells (CD45‐, SYTOX+) when the tumor cells were cocultured with PBMCs under HT‐DNA pretreatment (Figure 3E; and Figure S3E, Supporting Information). Overall, the above results illustrate that endocrine‐resistant breast cancer cells fail to mount the intrinsic death of cancer cells and its immune regulating function in response to the induction of dsDNA.

### 2.4 Hyperactivated AKT1 Kinase Interacts with TBK1 to Inhibit the Formation of STING/TBK1/IRF3 Trimers in Endocrine‐Resistant Breast Cancer

**Figure 4:**
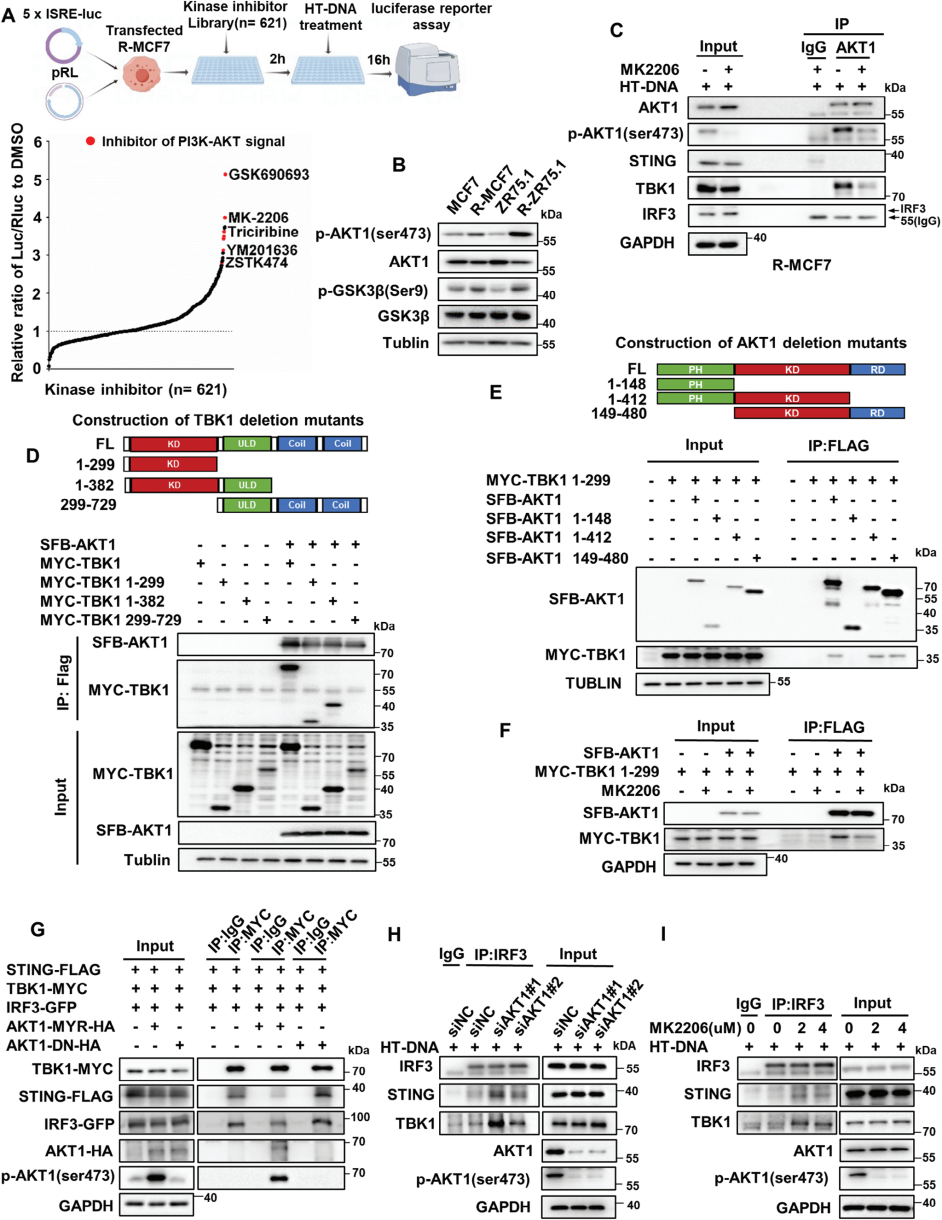
AKT1 interacts with TBK1 to inhibit the formation of STING/TBK1/IRF3 trimer in endocrine‐resistant breast cancer. A) Screening of the kinase inhibitor library revealed PI3K‐AKT signal to be a strong suppressor of STING signaling. B) MCF7/ZR75.1 cells and R‐MCF7/R‐ZR75.1 cells were harvested for western blot analysis of proteins in PI3K‐AKT pathway. C) R‐MCF7 cells were pretreated with 2 µm MK2206 for 2 h, then incubated with 2 µg mL^−1^ HT‐DNA for 6 h and harvested for coimmunoprecipitation assays. D) Domain mapping assays, performed by coimmunoprecipitation of SFB‐tagged full‐length (FL) AKT1 with serial truncations of TBK1 (upper), revealed that kinase domain of TBK1 was responsible and enough to bind AKT1 (bottom). E) Domain mapping assays, performed by coimmunoprecipitation of Myc‐tagged TBK1 kinase domain (1‐299) with serial truncations of AKT1 (upper), revealed that kinase domain of AKT1 was responsible and enough to bind TBK1 kinase domain (bottom). F) SFB‐tagged full‐length (FL) AKT1 and Myc‐tagged kinase domain of TBK1 were transfected into HEK‐293T cell for 48 h, then cells were treated with 2 µm MK2206 for 6 h and harvested for co‐immunoprecipitation assays, SFB‐AKT1 was immunoprecipitated with FLAG antibody. Cell lysates and IP were analyzed by western blot. G) HEK293T cells were transfected with AKT1‐MYR‐HA or AKT1‐DN‐HA and cotransfected with Myc‐TBK1, GFP‐IRF3, and FLAG‐STING for 48 h. Cells were lysed and Myc‐TBK1 was immunoprecipitated with Myc antibody. Cell lysates and IP were analyzed by western blot. H) siAKT1 was used to interfere the expression of AKT1 in R‐MCF7 cells for 48 h, then cells were treated with 2 µg mL^−1^ HT‐DNA for 12 h and harvested for coimmunoprecipitation assays. I) R‐MCF7 cells were pretreated with MK2206 for 2 h, then cells were treated with 2 µg mL^−1^ HT‐DNA for 12 h and harvested for coimmunoprecipitation assays. All data are representative of three independent experiments.

The molecular basis underlying inactivation of the cGAS‐STING pathway was then investigated. cGAMP, produced by cGAS, is recognized by STING located at the ER and then triggers STING activation. However, we found that the expression levels of cGAS and STING in MCF7/ZR75.1 cells were not significantly different from those in R‐MCF7/R‐ZR75.1 cells (Figure S4A, Supporting Information). Consistent with that, ELISA showed that there was no significant difference in the cGAMP of cell lysate between MCF7/ZR75.1 cells and R‐MCF7/R‐ZR75.1 cells when treated with HT‐DNA (Figure S4B, Supporting Information). We also found that HT‐DNA treatment significantly promoted STING transport from the ER to the Golgi in both ZR75.1 and R‐ZR75.1 cells, but there was no significant difference (Figure S4C, Supporting Information). To further explore the mechanism underlying the suppression of STING signaling in endocrine‐resistant breast cancer, we employed a kinase inhibitor library and assessed the role of individual kinase inhibitors in reversing activity of STING signaling. Reporter screening revealed that inhibitors of PI3K‐AKT pathway markedly activated STING signaling in endocrine‐resistant breast cancer (**Figure** **4**A; and Table S1, Supporting Information). Consist with that, GSEA showed that the PI3K‐AKT‐mTOR pathway was the only upregulated pathway in both tumor tissues of endocrine‐insensitive patients and endocrine‐resistant breast cancer cells (Figure S4D, Supporting Information). Accordingly, we found that the expression of p‐AKT1 and p‐GSK3β in R‐MCF7/R‐ZR75.1 cells was much higher than that in MCF7/ZR75.1 cells (Figure 4B). A coimmunoprecipitation assay showed that AKT1 mainly interacted with TBK1 rather than STING or IRF3 in R‐MCF7 cells (Figure 4C). To identify the domain of TBK1 that is responsible for its interaction with AKT1, we constructed several deletion mutants according to the conserved domains of TBK1. The results showed that AKT1 could interact with deletion mutant TBK1, while this interaction did not occur when TBK1 lacked the kinase domain (Figure 4D). Similarly, we constructed several deletion mutants according to the conserved domains of AKT1. The interaction between the TBK1 kinase domain and deletion mutants AKT1 did not occur when AKT1 lacked the kinase domain (Figure 4E), indicating that the TBK1 kinase domain and AKT1 kinase domain were essential for the interaction between TBK1 and AKT1. Moreover, we found that the interaction between the TBK1 kinase domain and AKT1 was significantly blunted by MK2206 (Figure 4F). The formation of the STING/TBK1/IRF3 trimer is essential for activation of the cGAS‐STING pathway.^[^ ^26^ ^]^ To determine how the AKT1/TBK1 interaction affects the formation of the trimeric complex, dominant‐negative AKT1 (AKT1‐DN) or constitutively activated AKT1 (AKT1‐MYR) was cotransfected with TBK1, STING, and IRF3 into HEK293T cells. Cotransfection of TBK1, STING, IRF3, and dominant‐negative AKT1 (AKT1‐DN) resulted in the coimmunoprecipitation of both STING and IRF3 with TBK1. However, constitutively activated AKT1 (AKT1‐MYR) expression disrupted the coimmunoprecipitation of both STING and IRF3 with TBK1 (Figure 4G). Furthermore, knockdown of AKT1 expression in R‐MCF7 cells significantly enhanced the coimmunoprecipitation of both STING and TBK1 with IRF3 (Figure 4H). Similarly, the coimmunoprecipitation of both STING and TBK1 with IRF3 was improved in R‐MCF7 cells when tumor cells were treated with MK2206 (Figure 4I). In summary, these results indicate that activated AKT1 can bind to TBK1 at the kinase domain and prevent TBK1 from activating IRF3 in endocrine‐resistant breast cancer.

### 2.5 Targeting AKT1 Reverses cGAS‐STING Pathway Activity in Endocrine‐Resistant Breast Cancer Cells

**Figure 5:**
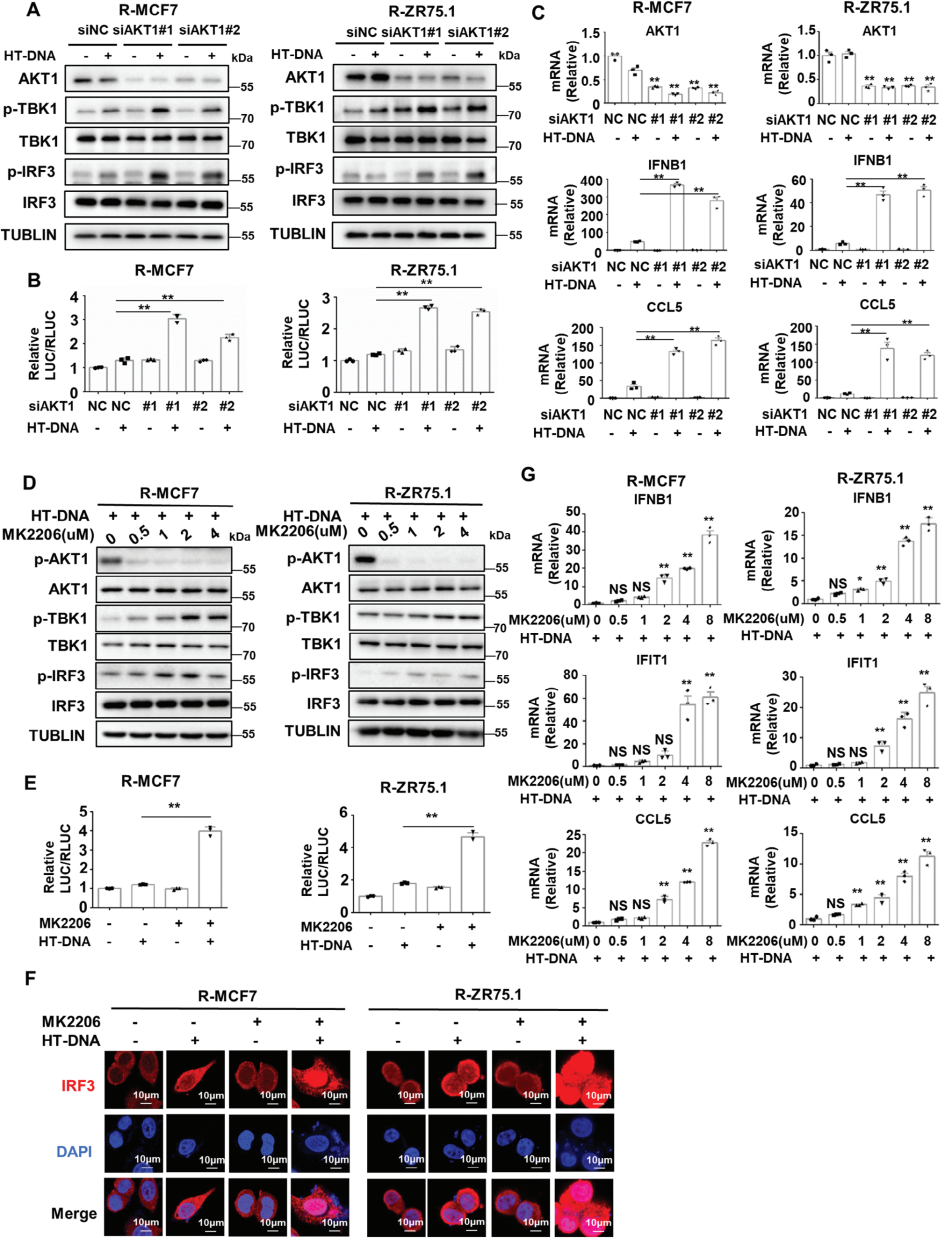
Targeting AKT1 reverses the activity of cGAS‐STING pathway in endocrine‐resistant breast cancer cells. A) Knockdown AKT1 in R‐MCF7/R‐ZR75.1 cells for 48 h, then cells were treated with 2 µg mL^−1^ HT‐DNA for 12 h and harvested for western blot analysis of proteins in cGAS‐STING pathway. B) A luciferase‐reporter assay with an IRF3‐responsive ISRE promoter stimulated by cGAS‐STING pathway was used in R‐MCF7/R‐ZR75.1 cells, after treated with 2 µg mL^−1^ HT‐DNA for 16 h, luciferase activity was detected (*n* = 3 biological independent samples). C) Knockdown AKT1 in R‐MCF7/R‐ZR75.1 cells for 48 h, then cells were treated with 2 µg mL^−1^ HT‐DNA for 12 h and harvested for RT‐qPCR analysis of AKT1 mRNA, IFNB1 mRNA, and CCL5 mRNA (*n* = 3 biological independent samples). D) R‐MCF7/R‐ZR75.1 cells were pretreated with MK2206 for 2 h, then cells were treated with 2 µg mL^−1^ HT‐DNA for 12 h and harvested for western blot analysis of proteins in cGAS‐STING pathway. E) A luciferase‐reporter assay with an IRF3‐responsive ISRE promoter stimulated by cGAS‐STING pathway was used in R‐MCF7/R‐ZR75.1 cells, after treated with 2 µg mL^−1^ HT‐DNA for 16 h, luciferase activity was detected (*n* = 3 biological independent samples). F) R‐MCF7/R‐ZR75.1 cells were pretreated with MK2206 for 2 h, then cells were treated with 2 µg mL^−1^ HT‐DNA for 12 h and harvested for immunofluorescence detection of IRF3 (red). G) R‐MCF7/R‐ZR75.1 cells were pretreated with MK2206 for 2 h, then cells were treated with 2 µg mL^−1^ HT‐DNA for 12 h and harvested for RT‐qPCR analysis of IFNB1 mRNA, IFIT1 mRNA, and CCL5 mRNA (*n* = 3 biological independent samples). *p*‐values were calculated by unpaired two‐tailed Student's *t*‐test, *p < 0.05; **p < 0.01; NS, not significant. All data are representative of three independent experiments.

We further confirmed whether targeting AKT1 could reverse the activity of the cGAS‐STING pathway in endocrine‐resistant breast cancer cells. The western blot results showed that knockdown of AKT1 significantly increased the phosphorylation of TBK1 and IRF3 when R‐MCF7/R‐ZR75.1 cells were treated with HT‐DNA (**Figure** **5**A). Moreover, a dual‐luciferase reporter assay showed that AKT1 knockdown markedly enhanced the HT‐DNA‐induced transcriptional activity of IRF3 in R‐MCF7/R‐ZR75.1 cells (Figure 5B). Correspondingly, more IRF3 was translocated into the nucleus after HT‐DNA treatment when AKT1 was knocked down in R‐MCF7/R‐ZR75.1 cells (Figure S5A, Supporting Information). Furthermore, we observed that AKT1 knockdown significantly increased the mRNA expression of IFNB1 and CCL5 when R‐MCF7/R‐ZR75.1 cells were transfected with HT‐DNA (Figure 5C). Consistent with AKT1 knockdown, MK2206 treatment significantly increased the HT‐DNA‐induced phosphorylation of TBK1 and IRF3 in R‐MCF7/R‐ZR75.1 cells (Figure 5D). In addition, MK2206 treatment promoted the transcriptional activity of IRF3 when R‐MCF7/R‐ZR75.1 cells were transfected with HT‐DNA (Figure 5E). Accordingly, more IRF3 was translocated into the nucleus after HT‐DNA treatment when R‐MCF7/R‐ZR75.1 cells were treated with MK2206 (Figure 5F). Moreover, RT‐qPCR analysis showed that MK2206 treatment markedly increased the HT‐DNA‐induced mRNA expression levels of IFNB1, IFIT1, and CCL5 in R‐MCF7/R‐ZR75.1 cells (Figure 5G). Furthermore, we employed capivasertib, an AKT inhibitor that has been approved by FDA, to detect its effect on the cGAS‐STING pathway, and the results showed that capivasertib could significantly improve HT‐DNA‐induced phosphorylation of TBK1 and IRF3 in endocrine resistant breast cancer cells (R‐MCF7/R‐ZR75.1) (Figure S5B, Supporting Information). In addition, capivasertib can also significantly increase the expression of IFNB1, IFIT1, and CCL5, downstream molecules of the cGAS‐STING pathway in endocrine resistant breast cancer (Figure S5C, Supporting Information), suggesting that capivasertib can also improve the activity of cGAS‐STING pathway in endocrine‐resistant breast cancer. Taken together, these results suggest that AKT1 is a crucial regulator of STING signaling in endocrine‐resistant breast cancer cells and that knockdown of AKT1 or targeting AKT1 by MK2206 could release the activity of STING signaling.

### 2.6 Inactivation of cGAS‐STING Signaling Forms a Positive Feedback Loop with Hyperactivated AKT1 to Promote Endocrine Resistance

**Figure 6:**
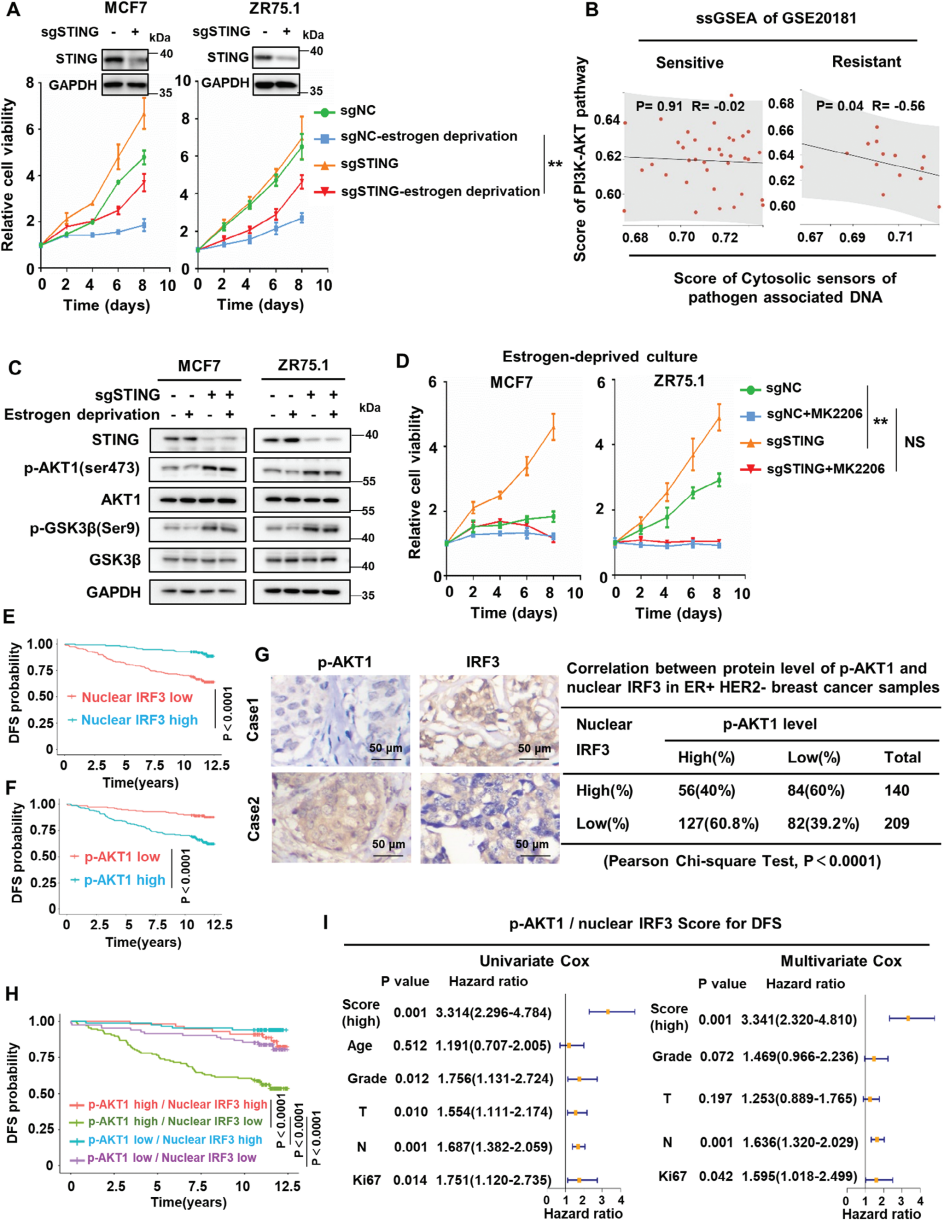
The positive feedback loop of inactivated STING signaling and hyperactivated AKT1 in ER+HER2– breast cancer. A) Cell proliferation assay for the growth rate of MCF7/ZR75.1 cells with or without STING depletion in standard medium or estrogen‐deprived medium. B) Correlation analysis between the enrichment score of cytosolic sensors of pathogen associated DNA pathway and PI3K‐AKT pathway based on ssGSEA of GES20181. C) MCF7/ZR75.1 cells with or without STING depletion were harvested for western blot analysis of proteins in PI3K‐AKT pathway. D) Cell proliferation assay for the growth rate of MCF7/ZR75.1 cells with or without STING depletion under MK2206 treatment in estrogen‐deprived medium. E) Kaplan–Meier plots of the DFS of patients, stratified by protein expression of nuclear IRF3. The *p* value was assessed using the log‐rank test (two‐sided). F) Kaplan–Meier plots of the DFS of patients, stratified by protein expression of p‐AKT1. The *p* value was assessed using the log‐rank test (two‐sided). G) The representative images for p‐AKT1 staining in two patients with nuclear IRF3 expression (left). Case 1 showed low expression of p‐AKT1 with high expression of nuclear IRF3. Case 2 showed high expression of p‐AKT1 with low expression of nuclear IRF3. The correlation of p‐AKT1 and nuclear IRF3 expression status in ER+HER2– breast cancer tissues (right). The relationship was assessed using Pearson's chi‐square test. H) Kaplan–Meier plots of the DFS of patients, stratified by protein expression of both p‐AKT1 and nuclear IRF3. The *p* value was assessed using the log‐rank test and further corrected with the Benjamini–Hochberg method (two‐sided). I) Univariate Cox regression analysis and multivariate Cox regression analysis regarding DFS for ER+HER2– breast cancer patients. *p*‐values were calculated by unpaired two‐tailed Student's *t*‐test, *p < 0.05; **p < 0.01; NS, not significant. All data are representative of three independent experiments.

To explore whether silencing of cGAS–STING signaling contributes to endocrine resistance, we depleted STING using specific single‐guide RNAs (sgRNAs) in parental MCF7 and ZR75.1 cells to construct STING signaling‐deficient cell lines. Estrogen‐deprived culture analysis showed that deficiency of STING signaling significantly increased the proliferation ability of parental MCF7 and ZR75.1 cells in estrogen‐deprived environment, promoting resistance to endocrine therapy (**Figure** **6**A). Subsequently, the potential mechanism of inactivated STING signaling promoting endocrine resistance was investigated. ssGSEA showed that the enrichment score of PI3K‐AKT pathway is negatively correlated with that of innate immune signal sensing cytosolic DNA in endocrine‐resistant breast cancer, not in endocrine‐sensitive breast cancer (Figure 6B). Combined with our findings that hyperactivated AKT1 blocked cGAS‐STING signaling, we proposed that inactivated STING signaling and hyperactivated AKT1 formed a positive feedback loop in endocrine‐resistant breast cancer. In fact, it has been proven that the upregulation of PI3K‐AKT‐mTOR signaling is associated with the endocrine resistance of breast cancer.^[^ ^3^, ^27^ ^]^ Consistently, deficiency of STING signaling in parental MCF7 and ZR75.1 cells enhanced the phosphorylation of AKT1 and GSK3β (Figure 6C), indicating activation of PI3K‐AKT signaling. In addition, estrogen‐deprived culture analysis showed that the increased proliferation ability by STING deletion can be inhibited by AKT inhibitors MK2206 in parental MCF7 and ZR75.1 cells (Figure 6D), confirming that STING deletion promotes endocrine resistance by up‐regulating activation of PI3K‐AKT signaling pathway. To further explore whether the hyperactivation of PI3K‐AKT signaling pathway mediated by STING deletion was related to type I interferon downstream of cGAS‐STING pathway, IFNAR1 was knocked down in MCF7 and ZR75.1 cells, and found that knockdown of IFNAR1 could also promote the phosphorylation of AKT1 and GSK3β, but further knockdown of STING on the basis of IFNAR1 knockdown could not further increase the phosphorylation of AKT and GSK3β (Figure S6A,B, Supporting Information), indicating that STING regulated the activation of PI3K‐AKT pathway through its downstream type I interferon.

We then assessed the clinical significance of p‐AKT1 and nuclear IRF3 in ER+ HER2– breast cancer patients who received adjuvant endocrine therapy. The protein levels of p‐AKT1 and nuclear IRF3 in 349 ER+HER2– breast cancer samples were evaluated by IHC (Figure S6C,D, Supporting Information). The Kaplan–Meier survival analysis showed that patients with high expression of nuclear IRF3 had longer DFS and OS than those in the low‐expression group (Figure 6E; and Figure S6E, Supporting Information), and the patients with high expression of p‐AKT1 had shorter DFS and OS compared to those in the low‐expression group (Figure 6F; and Figure S6F, Supporting Information). We also observed a significant negative correlation between the expression levels of p‐AKT1 and nuclear IRF3. The percentage of patients with high expression of p‐AKT1 among the patients with high expression of nuclear IRF3 (56/140 cases, 40%) was significantly lower than that among patients with low expression of nuclear IRF3 (127/209, 60.8%) (Figure 6G). Furthermore, we analyzed the prognostic value of combining p‐AKT1 and nuclear IRF3 protein levels in ER+ HER2– breast cancer patients. By combining p‐AKT1 high/low and nuclear IRF3 high/low expression, we separated patients into four groups and reperformed the survival analysis. Kaplan–Meier survival analysis showed that the DFS and OS of patients in the p‐AKT1^high^/nuclear IRF3^low^ group was significantly shorter than that of patients in the other three groups (Figure 6H; and Figure S6G, Supporting Information). Multivariate Cox regression analysis showed that the p‐AKT1/nuclear IRF3 score was an independent prognostic factor for the DFS and OS of ER+ HER2– breast cancer patients (Figure 6I; and Figure S6H, Supporting Information). Collectively, these results suggest that the positive feedback loop of inactivated STING signaling and hyperactivated AKT1 has important pathophysiological significance and clinical relevance to endocrine resistance in ER+ HER2– breast cancer patients.

### 2.7 The Combination of STING Agonists and AKT1 Inhibitors Enhances Immune Surveillance and Inhibits Tumor Growth of Endocrine‐Resistant Breast Cancer

**Figure 7:**
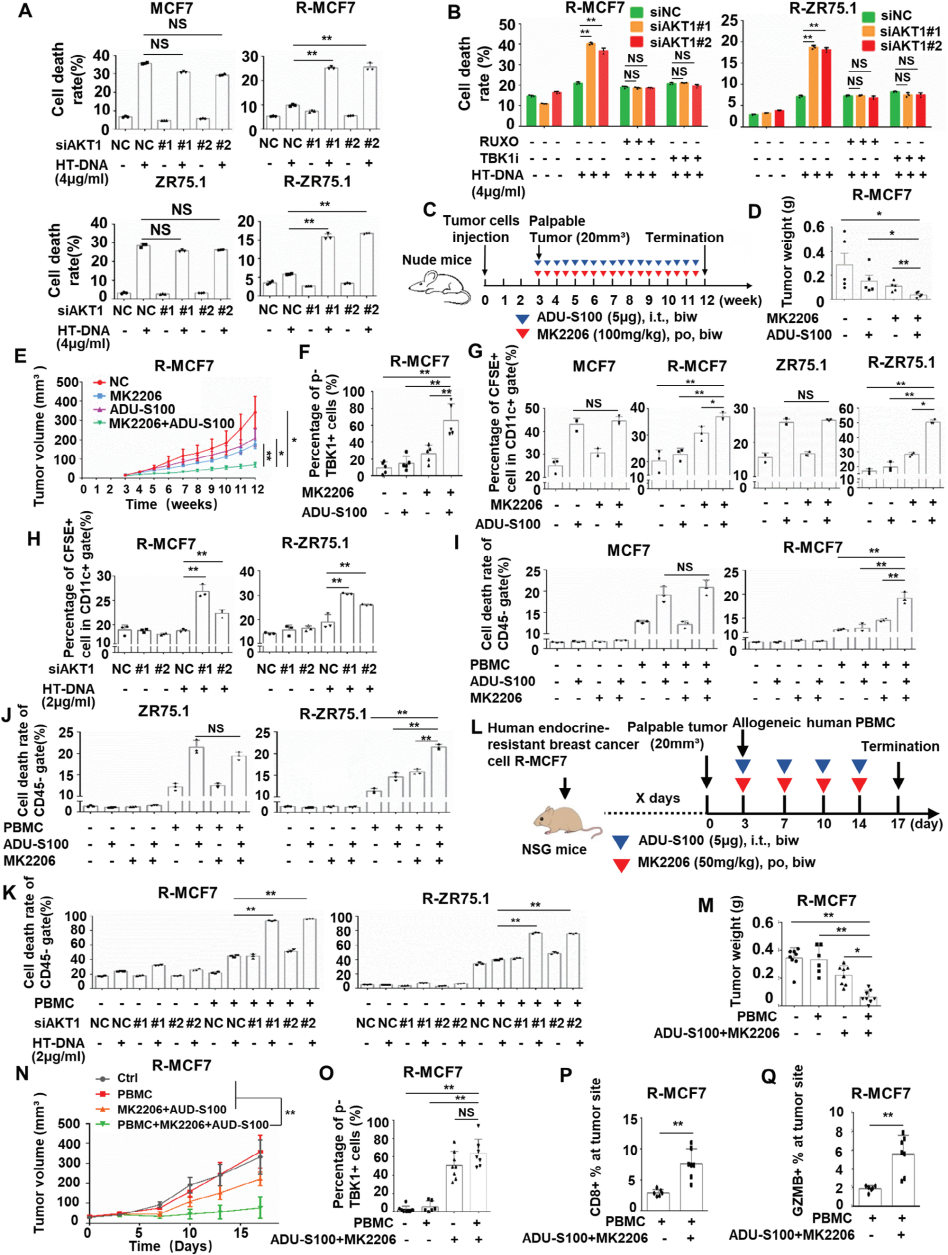
The combination of ADU‐S100 and MK2206 enhances immune surveillance to inhibit tumor growth of endocrine‐resistant breast cancer. A) Knockdown AKT1 in MCF7/ZR75.1 cells and R‐MCF7/R‐ZR75.1 cells for 48 h, then cells were treated with 4 µg mL^−1^ HT‐DNA for 24 h and harvested for Annexin V‐FITC/PI staining assay (*n* = 3 biological independent samples). B) Knockdown AKT1 in R‐MCF7/R‐ZR75.1 cells for 48 h, then cells were pretreated with 0.5 µm Ruxolitinib or 1 µm GSK8612 for 2 h. After that cells were treated with 4 µg mL^−1^ HT‐DNA for 24 h and harvested for Annexin V‐FITC/PI staining assay (*n* = 3 biological independent samples). C) Schematic overview of treatment dosage and schedule in nude mice. D) Graphical quantification of difference in weight of tumor at week 12 in each group. E) All nude mice were sacrificed at week 12 and graphical quantification represents the tumor growth rate in nude mice. F) Immunofluorescence detection of p‐TBK1 in tumor cells of tissues from different groups. G) MCF7/ZR75.1 cells and R‐MCF7/R‐ZR75.1 cells were pretreated with 2 µg mL^−1^ ADU‐S100 and 0.5 µm MK2206 for 24 h, then cancer cells were dyed by CFSE and cocultured with immature DC for 16 h and harvested for flow cytometry analysis of DC that engulfing the cancer cells (*n* = 3 biological independent samples). H) Knockdown AKT1 in R‐MCF7/R‐ZR75.1 cells for 48 h, then cells were pre‐treated with 2 µg mL^−1^ HT‐DNA for 6 h. After that cancer cells were dyed by CFSE and cocultured with immature DC for 16 h and harvested for flow cytometry analysis of DC that engulfing the cancer cells (*n* = 3 biological independent samples). I,J) MCF7/ZR75.1 cells and R‐MCF7/R‐ZR75.1 cells were pretreated with 2 µg mL^−1^ ADU‐S100 and 0.5 µm MK2206 for 24 h. After that, cancer cells were cocultured with PBMC (present with anti‐CD3, anti‐CD28, and IL‐2) for 72 h and harvested for flow cytometry analysis of cell death rate in cancer cells (*n* = 3 biological independent samples). K) siAKT1 was used to interfere the expression of AKT1 in R‐MCF7/R‐ZR75.1 cells for 8 h, then cells were pretreated with 2 µg mL^−1^ HT‐DNA for 6 h. After that, cancer cells were cocultured with PBMC (present with anti‐CD3, anti‐CD28, and IL‐2) for 72 h and harvested for flow cytometry analysis of cell death rate in cancer cells (*n* = 3 biological independent samples). L) Schematic overview of treatment dosage and schedule in NSG mice and humanized mice bearing R‐MCF7. M) Graphical quantification of difference in weight of tumor at day 17 in each group. N) Graphical quantification represents the tumor growth rate in NSG mice and humanized mice. O) Immunofluorescence detection of p‐TBK1 in tumor cells of tissues from different groups. P,Q) CD8+ T cell and GZMB+ cell infiltration of human endocrine‐resistant cancer R‐MCF7 in humanized mice treated with vehicle or MK2206 and ADU‐S100. *p*‐values were calculated by unpaired two‐tailed Student's *t*‐test, *p < 0.05; **p < 0.01; NS, not significant.

Currently, AKT inhibitor and STING agonists (STINGa) have been proved for clinical application or clinical trials.^[^ ^19^ ^]^ We proved that inactivated cGAS–STING signaling and hyperactivated AKT1 formed a positive feedback loop to mediate endocrine resistance in endocrine‐resistant breast cancer. Therefore, we speculated that the combination of STINGa and AKT1 inhibitors might block the positive feedback loop to suppress endocrine‐resistant tumors. In parental cells AKT1 was not hyperactivated, and HT‐DNA alone significantly increased cell death (PI staining positive) in MCF7/ZR75.1 cells, while knockdown of AKT1 did not further increase HT‐DNA‐induced cell death in MCF7/ZR75.1 cells. However, knockdown of AKT1 significantly increased cell death (PI staining positive) when R‐MCF7/R‐ZR75.1 cells were treated with HT‐DNA (**Figure** **7**A; and Figure S7A, Supporting Information). Moreover, either blocking the JAK‐STAT pathway with ruxolitinib or blocking the cGAS‐STING pathway with a TBK1 inhibitor abolished HT‐DNA induced cell death after AKT1 knockdown in R‐MCF7/R‐ZR75.1 cells (Figure 7B; and Figure S7B, Supporting Information). To validate the combined effect of AKT inhibitor and STINGa in vivo, the endocrine‐resistant breast cancer cell line R‐MCF7 was implanted in nude mice. After the tumors were palpable, mice were treated with MK2206 and ADU‐S100 as indicated (Figure 7C). Consistent with our observations in vitro, treatment with each single agent had a minimal effect, but the combined treatment with MK2206 and ADU‐S100 significantly improved tumor growth inhibition with synergistic antitumor effects (*Q* = 1.57),^[^ ^28^ ^]^ as confirmed by the growth curves of the xenograft tumor volumes and tumor weights (Figure 7D,E). Furthermore, double immunofluorescence staining showed that the phosphorylation of TBK1 in EpCam+ tumor cells in the combined treatment group was much higher than that in the control group and the monotherapy group (Figure 7F; and Figure S7C, Supporting Information). No obvious body weight loss was observed in the nude mice receiving the treatment (Figure S7D, Supporting Information).

Then, we investigated the influence of the combined treatment strategy on the antitumor immune response. In MCF7/ZR75.1 cells, ADU‐S100 monotherapy significantly increased the percentage of DCs that engulfed CFSE‐labeled tumor cells, but the effect of the combined treatment was not different from that of ADU‐S100 monotherapy. However, in R‐MCF7/R‐ZR75.1 cells, combining targeted inhibition of AKT1 (MK2206 or siAKT1) and activators of STING signaling (ADU‐S100 or HT‐DNA) significantly increased the percentage of DCs that engulfed CFSE‐labeled tumor cells much more than activators of STING signaling monotherapy (Figure 7G,H; and Figure S7E,F, Supporting Information). Furthermore, when MCF7/ZR75.1 cells were cocultured with PBMCs, ADU‐S100 monotherapy and the combined treatment increased the percentage of dead tumor cells to a similar extent. However, the combined treatment increased the percentage of dead tumor cells much more than activators of STING signaling monotherapy when R‐MCF7/R‐ZR75.1 cells were cocultured with PBMCs (Figure 7I–K; and Figure S7G,H, Supporting Information). We then investigated the combination effect in humanized mice bearing endocrine‐resistant tumors. We injected NSG mice with R‐MCF7 cells. Once tumor xenografts were established, we injected human PBMCs and monitored tumor growth (Figure 7L). In such models, tumor cells activate a response of human lymphocytes toward allogeneic antigens, which by nature are more immunogenic than tumor antigens. Despite this high immunogenicity, injecting PBMCs alone did not inhibit tumor growth (Figure 7M,N), indicating the presence of immunosuppressive mechanisms in endocrine‐resistant tumors. However, in the humanized mice model, the combination of MK2206 and ADU‐S100 significantly inhibited tumor growth, and its tumor inhibition effect in humanized mice was significantly stronger than that in non‐PBMCs reconstructed NSG mice, implying that part of the antitumor effect of the combination regimen was immune system dependent (Figure 7M,N). In addition, the combination regimen was well tolerated, and no significant weight loss was observed in the mice (Figure S7I, Supporting Information). Furthermore, the combination therapy significant enhanced the phosphorylation of TBK1 in EpCam+ tumor cells (Figure 7O; and Figure S7J, Supporting Information). Indeed, xenografts from combination treatment group contained increased amounts of human tumor infiltrating CD8+ lymphocytes and GZMB+ lymphocytes (Figure 7P,Q; and Figure S7K, Supporting Information). Together, these results support the rationale for combining treatment with STINGa and AKT1 inhibitors in endocrine‐resistant breast cancer, but not endocrine‐sensitive breast cancer.

## 3 Discussion

Currently, the overall efficacy of targeted therapy and immune checkpoint blockers (ICBs) for endocrine‐resistant breast cancer is unsatisfactory.^[^ ^5^, ^6^, ^29^ ^]^ The present study provides experimental and clinical evidence supporting a potentially interesting mechanism to mediate endocrine resistance from the perspective of innate immunity in endocrine‐resistant breast cancer. Our results show that the TBK1‐dependent activation of IRF3 is blunted in endocrine‐resistant breast cancer cells, thereby resulting in the suppression of cGAS‐STING signaling to mediate the endocrine resistance. Mechanistically, hyperactivated AKT1 binds to the kinase domain of TBK1, blocking the downstream signal transduction of the cGAS‐STING pathway. In addition, inactivation of STING signal reduces its suppression on phosphorylated AKT1. The formation of positive feedback loop between inactivated STING signaling and hyperactivated AKT leads to further amplification of AKT1 phosphorylation and suppressed STING signaling in endocrine‐resistant breast cancer, promoting its endocrine resistance. More importantly, we present a combination strategy of STING agonists and AKT1 inhibitors that could block the positive feedback loop to maximize the activation of cGAS‐STING signaling in endocrine‐resistant breast cancer cells, overcoming endocrine resistance.

Multiple mechanisms that are responsible for endocrine resistance have been proposed.^[^ ^30^ ^]^ Loss of the endocrine therapy target ER occurs in less than 10% of patients with endocrine‐resistant breast cancer.^[^ ^31^ ^]^ And most endocrine‐resistant breast cancers are driven by ligand‐independent reactivation of ER,^[^ ^32^ ^]^ mainly including the following four aspects: 1) gain‐of‐function mutation of ER; 2) compensatory interaction between ER and growth factor signaling, such as PI3K‐AKT signaling and MAPK signaling; 3) mutation of cell cycle‐related genes; and 4) changes in epigenetic modification.^[^ ^3^ ^]^ But the overall efficacy of targeted drugs based on these endocrine‐resistant mechanisms for endocrine‐resistant breast cancer is not satisfactory. At present, immune checkpoint inhibitor therapy has shown promising efficacy in a variety of cancers but failed in endocrine‐resistant breast cancer.^[^ ^6^, ^33^ ^]^ Current understanding of the role of the immune microenvironment in endocrine‐resistant breast cancer is limited. In this study, we revealed a novel endocrine‐resistant mechanisms that cGAS‐STING signaling interacted with AKT1, forming a positive feedback loop to promote the resistance to endocrine therapy. On one hand, inactivation of cGAS‐STING signaling reduced the production of type I interferon from endocrine‐resistant cancer cells. Due to the powerful antitumor effect of type I interferon in immune microenvironment, the blockade of cGAS‐STING signaling in endocrine‐resistant breast cancer promoted the tolerance to dsDNA‐induced cell death and inhibited the maturation of DCs and the cytotoxicity of PBMC immune cells, which could promote the formation of immunosuppressive microenvironment, tending to “cold” tumor. Mechanistically, hyperactivated AKT1 blocked the formation of STING/TBK1/IRF3 trimer in endocrine‐resistant breast cancer. On the other hand, inactivated cGAS‐STING signaling could in turn increase the phosphorylation of AKT1 to enhance the proliferation of endocrine‐resistant breast. Thus, the formation of positive feedback loop between inactivated cGAS‐STING signaling and hyperactivated AKT1 is a crucial determinant of endocrine resistance by mediating immunosuppressive microenvironment and promoting tumor proliferation. Furthermore, the positive feedback loop was demonstrated in tumor tissue from ER+HER2– breast cancer patients by IHC analysis. This study first revealed a novel molecular mechanism of endocrine resistance, which is related to innate immunity. To a certain extent, our discovery explains the poor efficacy of PD‐1 antibody plus chemotherapy in a clinical trial (NCT03051659) for endocrine‐resistant ER+HER2– breast cancer.^[^ ^6^ ^]^

cGAS‐STING signaling undoubtedly plays an important, centralized role in the immune‐mediated clearance of malignant cells; thus, several STING agonists have been developed and entered phase II clinical trials for antitumor therapy.^[^ ^18^ ^]^ Moreover, with the advancement of nanotechnology, an increasing number of nanoparticles have been developed to activate the cGAS‐STING pathway for antitumor therapy.^[^ ^34^, ^35^, ^36^, ^37^, ^38^ ^]^ In this study, we found that STING agonist monotherapy could effectively active the cGAS‐STING pathway in endocrine‐sensitive breast cancer to enhance antitumor immunity. The significant effect of STING monotherapy may be attributed to the fact that AKT1 is not hyperactivated in endocrine‐sensitive breast cancer, and the cGAS‐STING pathway is intact. However, in endocrine‐resistant breast cancer, STING agonist monotherapy failed to active the STING signaling, due to the positive feedback of inactivated STING signaling and hyperactivated AKT1 strengthening the suppression of cGAS‐STING pathway by AKT1. We found that targeted inhibition of AKT1 can release TBK1 protein from AKT1 kinase, thereby releasing the activity of the cGAS‐STING pathway in endocrine‐resistant breast cancer cells, indicating that combining STING agonists with AKT1 inhibitors could maximizes the activation of the cGAS‐STING pathway. Moreover, the combination treatment could block the positive feedback loop of suppressed STING signaling and hyperactivated AKT1 to overcome endocrine resistance. Nowadays, cancer treatment has entered the era of personalized and precision medicine.^[^ ^39^, ^40^, ^41^ ^]^ Our study revealed that endocrine‐resistant breast cancer patients with p‐AKT1^high^ and nuclear IRF3^low^ by immunohistochemistry might be a suitable population to receive a combination regimen of AKT inhibitors and STING agonists. As expected, the results of in vitro experiments and in vivo experiment of humanized mice models showed that the combination of the AKT inhibitor MK2206 and the STING agonist ADU‐S100 powerfully boosted the antitumor immunity response and inhibited the tumor growth of endocrine‐resistant breast cancer, not in endocrine‐sensitive breast cancer. Although previous studies have demonstrated that AKT inhibitors significantly inhibit breast cancer in animal models, the dose of AKT inhibitors in animal models is high (480 mg kg^−1^, qw).^[^ ^42^ ^]^ Dose restriction toxicity observed in clinical trials greatly limits the efficacy of AKT inhibitors in patients.^[^ ^43^ ^]^ So, it is reasonable to reduce toxicity and improve efficacy by reducing the dose of AKT inhibitors through a combination treatment strategy. In the present study, we found that combining STING agonists with AKT1 inhibitors could reduce the dose of AKT1 inhibitors to attenuate side effects or other toxicities. On the other hand, knockdown AKT1 or targeting AKT1 by MK2206 could release the activity of STING signaling in endocrine‐resistant breast cancer, and on this basis, adding STING agonists maximizes the activation of the cGAS‐STING pathway and blocks the positive feedback loop, which on the one hand changes endocrine‐resistant breast cancer from cold tumors to hot tumors to comprehensively enhance the antitumor immune response and on the other hand reduces the activation of PI3K‐AKT signal to inhibit tumor proliferation, both of which work together to overcome endocrine resistance.

In summary, our study confirms that hyperactivated AKT1 in endocrine‐resistant breast cancer can interact with TBK1 to block the activation of cGAS‐STING signaling. Notably, inactivation of STING signaling forms a positive feedback loop with hyperactivated AKT1 to promote endocrine resistance. Due to the importance of the positive feedback loop in endocrine resistance, this study provides a clinically applicable strategy consisting of STING agonists combined with AKT inhibitors to improve current antitumor therapy for endocrine‐resistant breast cancer patients.

## 4 Experimental Section

### Cell Culture and Compounds

Human MCF7, ZR75.1, and HEK293T were bought from the American Type Culture Collection (Manassas, VA) and maintained in Dulbecco's modified Eagle's medium (DMEM) supplemented with 10% fetal bovine serum (FBS, Gibico) at 37 °C under 5% CO_2_. Endocrine‐resistant cell lines were generated as recently described.^[^ ^44^ ^]^ All the cells were authenticated using short‐tandem repeat profiling and tested negative for mycoplasma contamination. Compounds HT‐DNA (D6898, Sigma), Poly(dA:dT)/LyoVecTM (tlrl‐patc, InvivoGen), ADU‐S100 (HY‐12885B‐1 mg, MCE), MK2206 (T1952, Topscience), LIPOFECTAMINE 3000 (L3000015, Invitrogen), Ruxolitinib (T1829, Topscience), GSK8612 (T5540, Topscience), Capivasertib (T1920, Topscience) were obtained commercially.

### Spatial Transcriptomics Using Stereo‐Seq

For spatial transcriptomics experiments of 4 samples of primary breast cancer, Stereo‐seq chips were used.^[^ ^45^ ^]^ These four early ER+HER2– breast cancer patients received endocrine therapy after surgery and chemotherapy according to NCCN guideline.^[^ ^46^ ^]^ Endocrine resistance is defined as the occurrence of tumor recurrence during endocrine therapy. Endocrine sensitivity was defined as the absence of tumor recurrence during and after endocrine therapy. According to this definition, 2 patients were defined as endocrine resistant and 2 patients were defined as endocrine sensitive. The clinical characteristics of these 4 patients were shown in Table S2 (Supporting Information). To ensure the efficiency of spatial transcriptomic analysis, the tissue slices to be tested are required to reach a sufficient area. If the patient's fresh tumor tissue was relatively small, it would be cut two slices of the patient's tumor tissue, and then combine the two tissue slices together as one sample to reach a sufficient area. SPOTlight v0.1.7 package was applied to deconvolute cell type, and a published breast cancer scRNA dataset.^[^ ^47^ ^]^ was used as the reference input to SPOTlight. For scoring and statistical analysis, ssGSEA scoring of the pathway gene sets was performed on tumor region using GSVA package.^[^ ^48^ ^]^ The detail of Spatial transcriptomics is described in the Supporting Information.

### Immunoblot and Immunoprecipitation

The process of immunoblot and immunoprecipitation was described previously.^[^ ^49^, ^50^ ^]^ The details of immunoblot and immunoprecipitation are described in the Supporting Information. Antibodies used in immunoblot and immunoprecipitation were listed in Table S3 (Supporting Information).

### Immunofluorescence

The process of Immunofluorescence was described previously.^[^ ^51^ ^]^ The detail of Immunofluorescence is described in the Supporting Information.

### Kinase Inhibitor Library Screen

The kinase inhibitor library containing 621 compounds was purchased from Selleck (L1200). Cells were seeded in 24‐well plates at a density of 1 × 10^5^ cells per well. On the second day, cells were transfected with the 5 × ISRE reporters (100 ng) bearing an ORF coding for the Firefly luciferase along with the pRL‐Luc with the Renilla luciferase ORF as the internal control for transfection. Briefly, at 12 h post transfection, the cells were pretreated with the indicated inhibitors for 2 h, then 2 µg mL^−1^ HT‐DNA were transfected into cells and lysed in a passive lysis buffer (Promega) 16 h after HT‐DNA transfection. The luciferase assays were performed using a dual luciferase assay kit (Promega), quantified with POLARstar Omega (BMG Labtech), and normalized to the internal Renilla‐luciferase activity.

### CRISPR–Cas9‐Mediated Gene Knockout

We used CRISPR–Cas9 technology to knock out STING. sgRNA was cloned into the empty backbone of lenti‐CRISPR v2. The following sgRNA sequence was used: sgSTING, 5′‐GCAGGCACTCAGCAGAACCA‐3′. Plasmids containing the sgRNA sequences were transfected into HEK293T cells with the psPAX2 packaging plasmid and pMD2.G VSV‐G envelope‐expressing plasmid. The virus was collected for 72 h, and then MCF7, ZR75.1 and other cells were infected with virus and 0.8 µg mL^−1^ polybrene and selected with puromycin (2 µg mL^−1^; Invivogen) for 3 d.

### Immunohistochemical Staining

The process of immunohistochemical staining was described previously.^[^ ^51^, ^52^ ^]^ Briefly, for human ER+ HER2– breast cancer analysis, 349 paraffin blocks of human ER+ HER2– breast lesions were selected for this study. These samples were histopathologically and clinically diagnosed as ER+ HER2– breast cancer at the Sun Yat‐sen University Cancer Center. These samples were selected from patients with available follow‐up data, no distant metastasis, and no neoadjuvant therapy history. All samples used in this study were approved by the medical ethics committee of Sun Yat‐sen University Cancer Center. For evaluation of p‐AKT1 staining, a staining index was adopted by multiplying the score for the percentage of positive tumor cells by the intensity score, which obtained as the intensity staining (0, no staining; 1, weak; 2, moderate; 3, strong) and the percentage of positive cells (0, <10%; 1, 10%–25%; 2, 26%–50%; 3, 51%–75%; 4, 76%–100%). Sections with a final score <4 were considered as low p‐AKT1 expression, whereas sections with a final score > = 4 were considered as high p‐AKT1 expression. For evaluation of IRF3 nuclear staining, a staining index was adopted by multiplying the score for the percentage of positive nuclear staining tumor cells and the intensity score, which obtained as the intensity staining (0, no staining; 1, weak; 2, moderate; 3, strong) and the percentage of positive cells (0, <10%; 1, 10%–25%; 2, 26%–50%; 3, 51%–75%; 4, 76%–100%). Sections with a final score <5 were considered as nuclear IRF3 high, whereas sections with a final score > = 5 were considered as nuclear IRF3 low. To analyze the prognostic value of combining p‐AKT1 and nuclear IRF3 protein levels, the composite scores of p‐AKT1 expression > = 4 and nuclear IRF3 > = 5 were assigned as “p‐AKT1^high^/ nuclear IRF3^high^” group, p‐AKT1 expression <4, and nuclear IRF3 expression <5 were assigned as “p‐AKT1^low^/ nuclear IRF3^low^” group, p‐AKT1 expression > = 4, and nuclear IRF3 expression <5 were assigned as “p‐AKT1^high^/ nuclear IRF3^low^” group, p‐AKT1 expression <4, and nuclear IRF3 expression > = 5 were assigned as “p‐AKT1^low^/ nuclear IRF3^high^” group. To quantify the combined value of p‐AKT1 and nuclear IRF3, a p‐AKT1/nuclear IRF3 score was adopted by summing the score for p‐AKT1(0, p‐AKT1 low; 1, p‐AKT1 high) and the score for nuclear IRF3 (0, nuclear IRF3 high; 1, nuclear IRF3 low).

### Human Xenografts in NSG Mice Reconstituted with Human Lymphocytes

3‐ to 4‐week‐old female NSG mice were obtained from Gempharmatech‐GD Company. Handling of mice and experimental procedures were conducted in accordance with national and institutional guidelines for animal care. NSG mice were injected subcutaneously with 5  ×  10^6^ R‐MCF7 cells. After tumor appearance, tumors were measured, mice were randomized on the basis of tumor size and injected or not with 1  ×  10^7^ human allogeneic HLA‐mismatched PBMCs intravenously. The human allogeneic HLA‐mismatched PBMCs was purchased from Shanghai Milestone Biotechnologies (P123020911C). The MK2206 was given by oral gavage at dose of 50 mg kg^−1^ for each NSG mouse. ADU‐S100 was given by intratumor injection at dose of 5 µg for each tumor. The frequency of treatment was twice a week. Tumor volumes and body weight of mice were observed. Volumes were calculated by the formula: 0.5 × A × B2 in millimeters, where A is the length and B is the width. After nude mice were sacrificed, the tumor tissues were excised and weighed.

### Statistical Analysis

R software (version 4.0.3) and GraphPad Prism 6.0.1 (GraphPad, La Jolla, CA) were used to conduct the statistical analyses. Survival curves were plotted by the Kaplan–Meier method in R software. The cutoff values for survival analysis of luminal‐A breast cancer patients in TCGA were established based on the best cutoff values in the function “surv_cutpoint” of “survminer” package. The *p* values were assessed using the log‐rank test and further corrected with the Benjamini–Hochberg method. Univariate Cox proportional hazards regression was carried out to identify HR (hazard ratios) and 95% CI (Confidence intervals). Multivariate analysis was used to determine independent prognostic factors using a Cox proportional hazards regression model. The results presented as the mean ± SD were analyzed by an unpaired Student's *t*‐test, or one‐way ANOVA with Dunnett's multiple comparisons test, or one‐way ANOVA with Tukey's multiple comparisons test, or Wilcoxon matched‐pairs signed‐rank test using GraphPad Prism. To evaluate the effect of combined treatment in vivo experiment, *Q* value method of Zhengjun jin was adopted.^[^ ^28^ ^]^ value > 1.15 was synergistic; 0.85–1.15 was additive; <0.85 was antagonistic. All the statistical tests were two‐sided, *p* < 0.05 was considered statistically significant.

### Ethical Statement

The research complied with all relevant ethical regulations. All animal procedures were approved by the Experimental Animal Ethics Committee of Sun Yat‐sen University Cancer Center (L102012023220Q). All samples from patients in this study were obtained with the informed consent of the patients and approved by the medical ethics committee of Sun Yat‐sen University Cancer Center (G2023‐161‐01).

## Conflict of Interest

The authors declare no conflict of interest.

## Author Contributions

K.‐M.Z., D.‐C.Z., Z.‐Y.L., and Y.W. contributed equally to this work. R.D., J.T., X.F.Z., and K.M.Z. designed the research. K.M.Z., D.C.Z., Z.Y.L., and Y.W. performed experiments and/or analyzed data. D.C.Z., Y.W., J.N.L., T.D., Y.H.C., Q.C.Y., Q.S.C., R.Z.C., Z.X.Z., J.L.S., B.X.H., H.L.Z., and G.K.F. provided advice on experiments. R.D., J.T., and X.F.Z. obtained funding and supervised the research. K.M.Z. and R.D. wrote the paper. K.M.Z., D.C.Z., Z.Y.L., and Y.W. are co‐first authors; authorship order reflects the degree to which authors drove key developments in the work.

The transcriptome data and clinical data of luminal‐A breast cancer patients were downloaded from the Cancer Genome Atlas (TCGA) database (https://portal.gdc.cancer.gov/repository).

The human sequence data generated in this study is uploaded to the China National Center for Bioinformation‐National Genomics Data Center (CNCB‐NGDC, https://ngdc.cncb.ac.cn/) and the data storage code is OMIX005632. The human sequence data that support the findings of this study have also been deposited into CNGB Sequence Archive (CNSA).^[^ ^53^ ^]^ of China National GeneBank DataBase (CNGBdb).^[^ ^54^ ^]^ with accession number CNP0005631.

## Supporting information

Supporting Information

## Data Availability

The data analyzed in this study were obtained from Gene Expression Omnibus (GEO) at GSE20181 and GSE75971.
