# Supplementary material for: Inactivated cGAS‐STING Signaling Facilitates Endocrine Resistance by Forming a Positive Feedback Loop with AKT Kinase in ER+HER2– Breast Cancer
Source: Adv Sci (Weinh). 2024 Jul 18;11(35):2403592. doi: 10.1002/advs.202403592 (PMC11425221; doi:10.1002/advs.202403592)
Supplement: Supplementary file 1 — Supporting Information [file ADVS-11-2403592-s001.docx]

**Inactivated cGAS–STING Signaling Facilitates Endocrine Resistance by Forming a Positive Feedback Loop with AKT Kinase in ER+HER2- Breast Cancer**

Kai-Ming Zhang#, De-Chang Zhao#, Ze-Yu Li#, Yan Wang#, Jian-Nan Liu, Tian Du, Ling Zhou, Yu-Hong Chen, Qi-Chao Yu, Qing-Shan Chen, Rui-Zhao Cai, Zi-Xuan Zhao, Jia-Lu Shan, Bing-Xin Hu, Hai-Liang Zhang, Gong-Kan Feng, Xiao-Feng Zhu, Jun Tang, Rong Deng^*^

K.-M. Zhang, D.-C. Zhao, Y. Wang, T. Du, L. Zhou, Y.-H. Chen, Q.-S. Chen, R.-Z. Cai, Z.-X. Zhao, J.-L. Shan, B.-X. Hu, H.-H. Zhang, G.-K. Feng, X.-F. Zhu, J. Tang, R. Deng. State Key Laboratory of Oncology in South China, Guangdong Provincial Clinical Research Center for Cancer, Collaborative Innovation Center for Cancer Medicine, Guangdong Key Laboratory of Nasopharyngeal Carcinoma Diagnosis and Therapy, Sun Yat-sen University Cancer Center, Guangzhou, 510060, China

K.-M. Zhang, D.-C. Zhao, Y. Wang, T. Du, Q.-S. Chen, R.-Z. Cai, Z.-X. Zhao, J. Tang. Department of Breast Oncology, Sun Yat-sen University Cancer Center, Guangzhou, 510060, China

Z.-Y. Li, Q.-C. Yu. BGI Research, Shenzhen, 518083, China.

Z.-Y. Li, Q.-C. Yu. College of Life Sciences, University of Chinese Academy of Sciences, Beijing, 100049, China.

J.-N. Liu. Department of Oncology, The Afﬁliated Yantai Yuhuangding Hospital of Qingdao University, Yantai, Shangdong, 264000, China.

# K.-M. Zhang, D.-C. Zhao, Z.-Y. Li, Y. Wang, contributed equally to this work.

* Correspondence and requests for materials should be addressed to R.D. (dengrong@sysucc.org.cn)

Figure. S1.

**
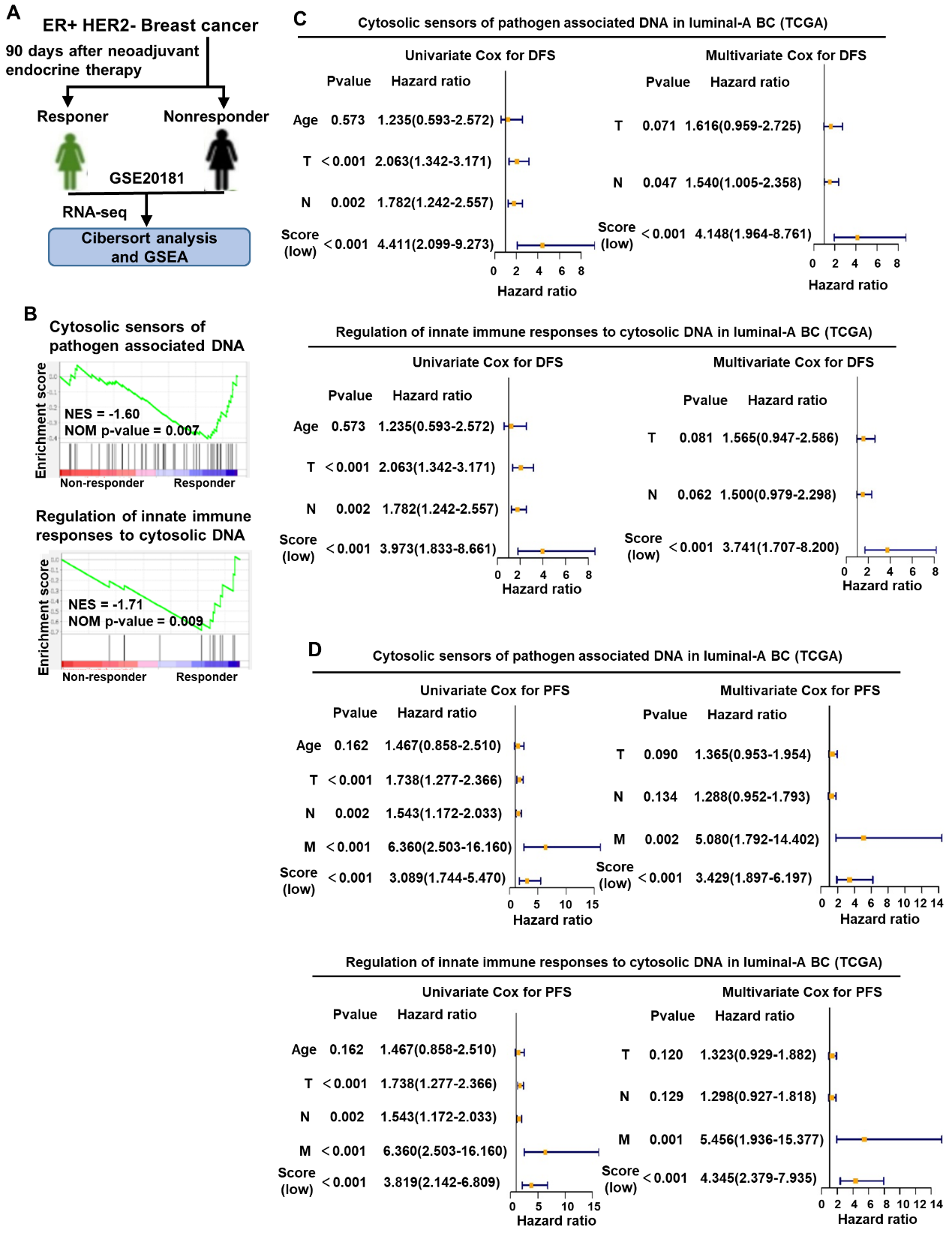
**

**
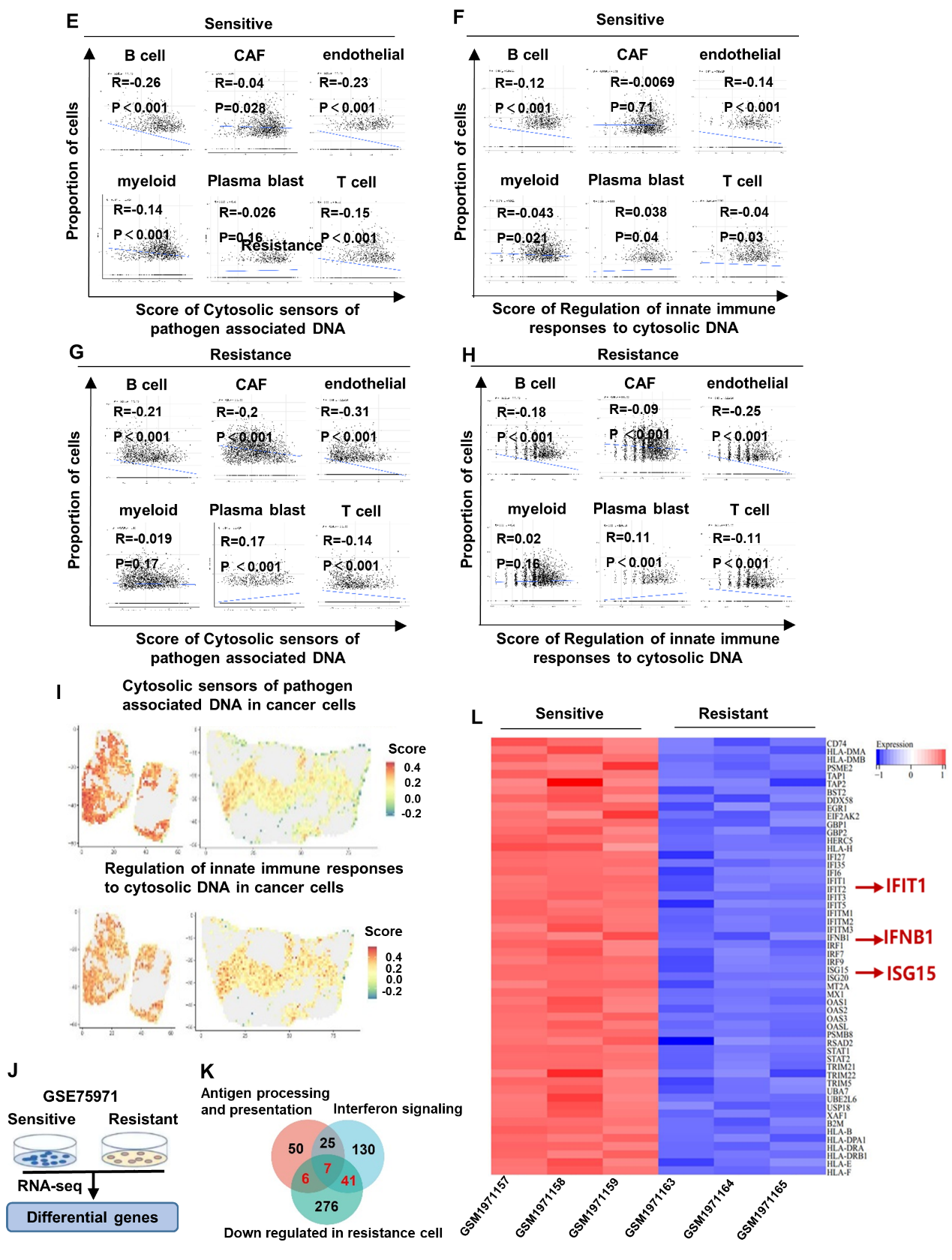
**

**Figure. S1. Innate immune signaling sensing cytosolic DNA were downregulated in endocrine-resistant breast cancer cell.** **A.** Illustration showing process for RNA-seq analysis of GSE20181 data set. **B.** Enrichment plot of cytosolic sensors of pathogen associated DNA pathway and regulation of innate immune responses to cytosolic DNA pathway based on GSEA analysis in GSE20181 data set. **C.** Univariate Cox regression analysis and multivariate Cox regression analysis regarding DFS for patients with Luminal-A breast cancer using the TCGA database. **D.** Univariate Cox regression analysis and multivariate Cox regression analysis regarding PFS for patients with Luminal-A breast cancer using the TCGA database. **E, F, G, H.** Pearson correlation analysis for the correlation between the abundance of different cell types and the enrichment score of innate immune signal sensing cytosolic DNA. **I.** Visualization of the spatial enrichment of innate immune signal sensing cytosolic DNA in tumor cells. **J.** Illustration showing process for RNA-seq analysis of GSE75971 data set. Venn diagram **(K)** and heatmap **(L)** showing 54 IRGs comprised of 48 interferon signaling genes and 13 antigen presentation and processing genes that were down-regulated in endocrine-resistant cells compared to parental cells (log fold cutoff >1, P < 0.05).

Figure. S2.

**
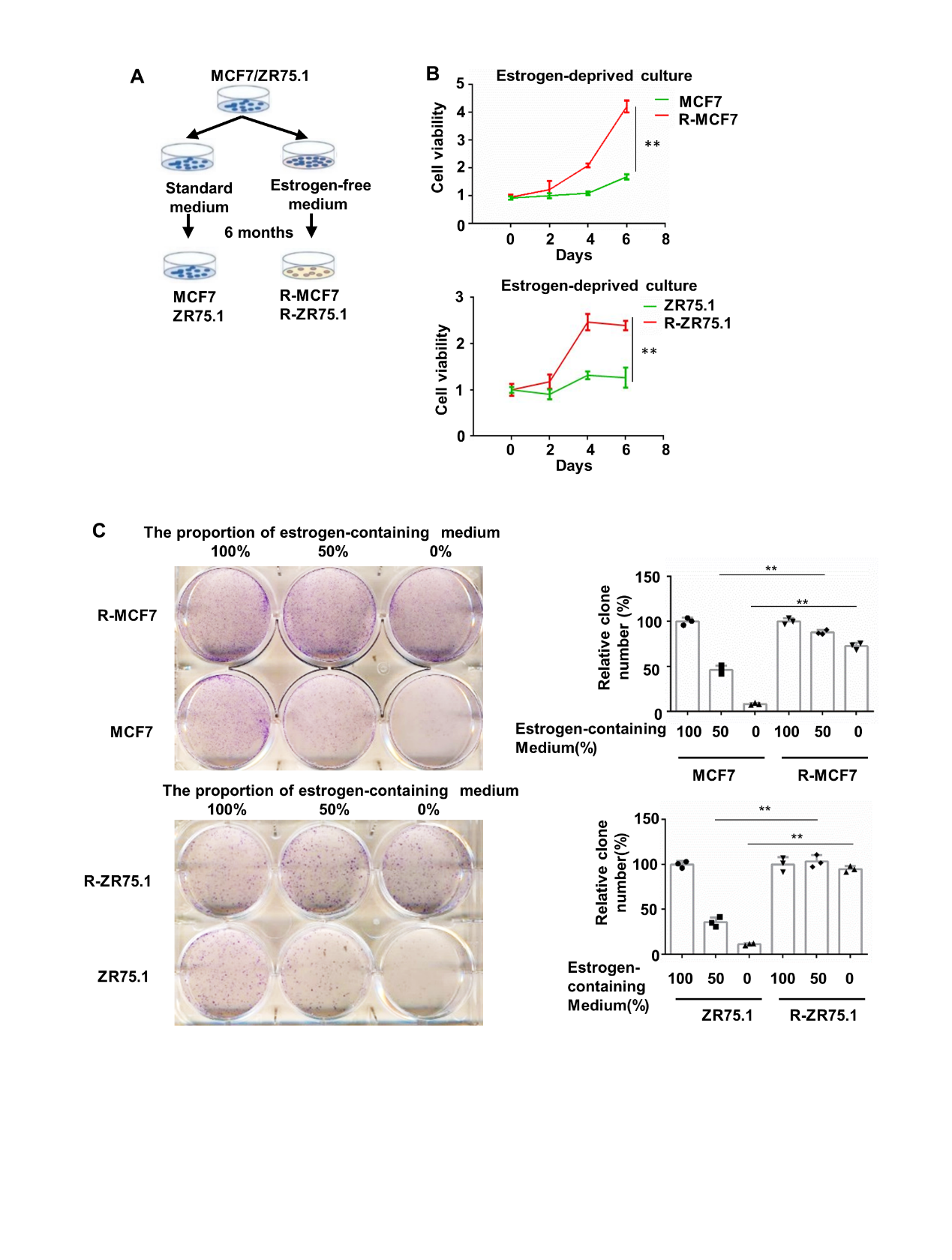
**

**Figure. S2. Construction of endocrine therapy resistant breast cancer cell lines by long-term estrogen deprivation. A.** Schematic of process for the construction of endocrine resistant R-MCF7/R-ZR75.1 cell lines. **B.** Cell proliferation assay for the growth rate of MCF7/ZR75.1 cells and R-MCF7/R-ZR75.1 cells in estrogen-deprived medium. **C.** Clonal formation assay for MCF7/ZR75.1 cells and R-MCF7/R-ZR75.1 cells in different proportions of estrogen-deprived medium.

Figure. S3.

**
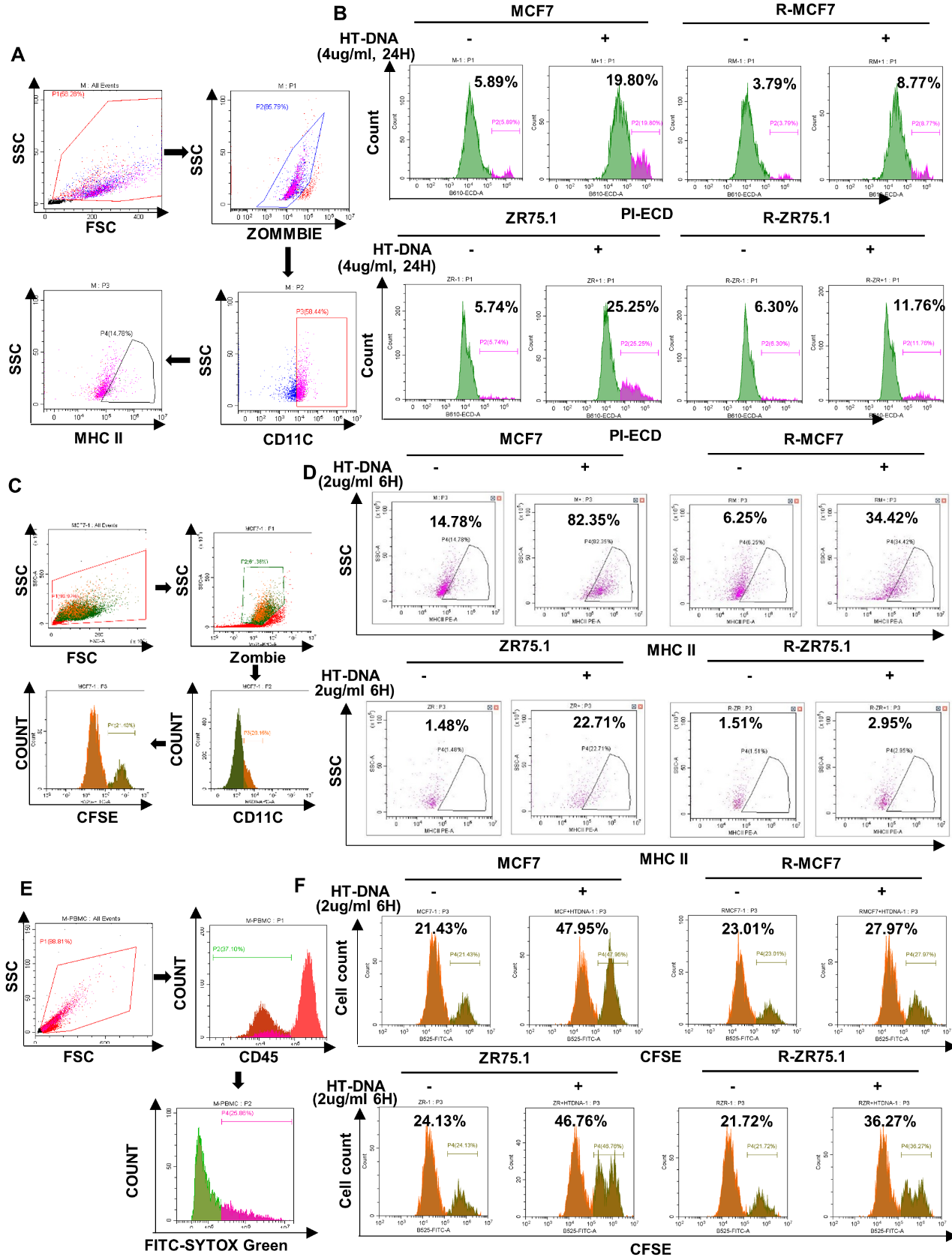
**

**Figure S3. Inactivation of cGAS-STING pathway in endocrine-resistant breast cancer mediates immune escape. A.** Gating strategy of flow cytometry to measure the DC mature marker, MHC II. **B.** Flow cytometry measuring HT-DNA induced cell death of MCF7/ZR75.1 cells and R-MCF7/R-ZR75.1 cells. **C.** Gating strategy of flow cytometry to measure the DCs that engulfing the CFSE dyed cancer cells. **D.** Flow cytometry measuring the DC mature marker, MHC II, when co-cultured with HT-DNA-treated MCF7/ZR75.1 cells and R-MCF7/R-ZR75.1 cells. **E.** Gating strategy of flow cytometry to measure the PI+ tumor cells when co-cultured with PBMCs. **F.** Flow cytometry measuring the DCs that engulfing the CFSE dyed cancer cells.

Figure. S4.

**
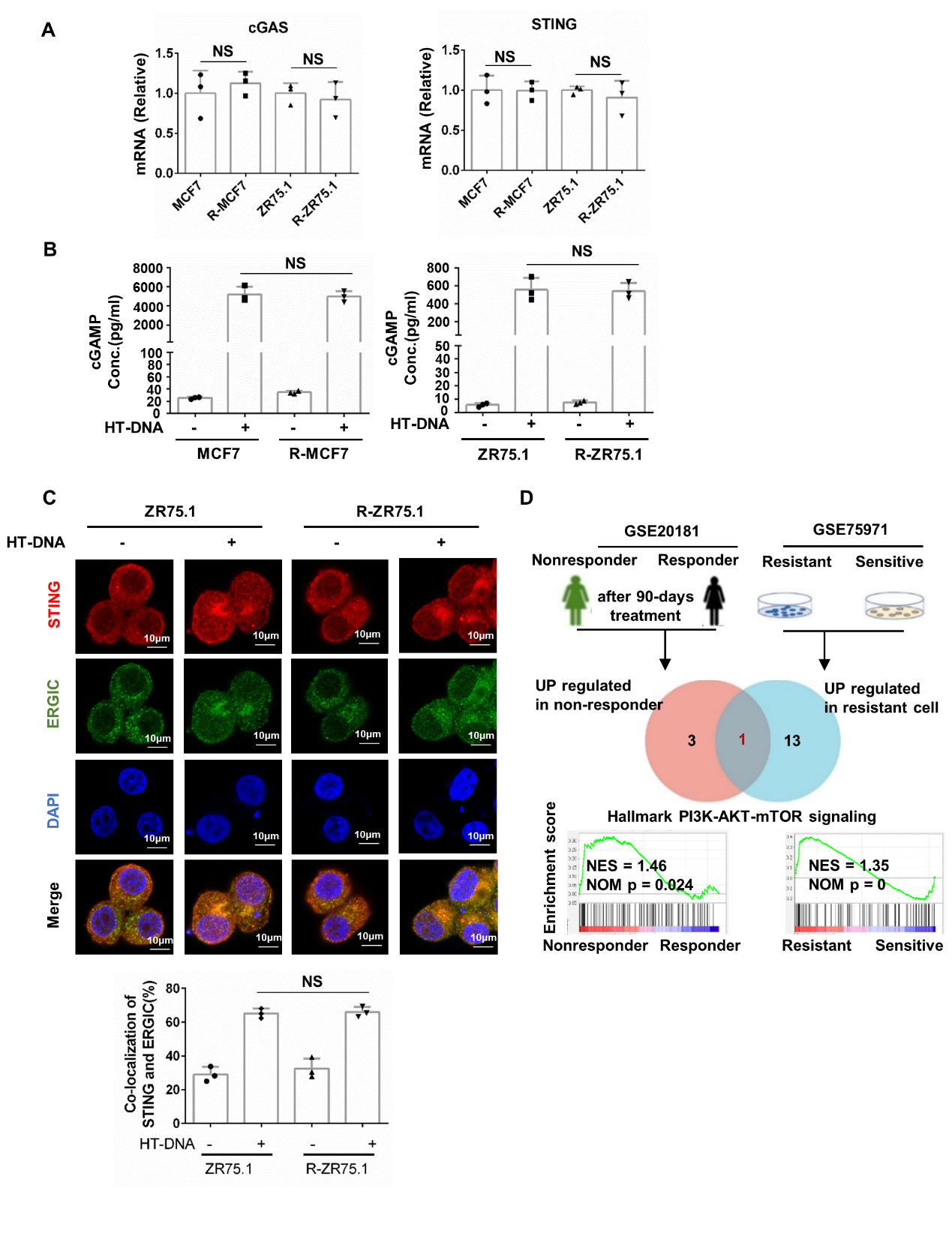
**

**Figure. S4. The production of cGAMP and the transportation of STING are not suppressed in endocrine-resistant cells. A.** RT-qPCR to test the mRNA expression level of cGAS and STING in MCF7/ZR75.1 cells and R-MCF7/R-ZR75.1 cells. **B.** MCF7/ZR75.1 cells and R-MCF7/R-ZR75.1 cells were treated with 2 ug/mL HT-DNA for 6 h and harvested for ELISA detection of cGAMP in cell lysate. **C.** Confocal analysis of the colocalization of STING (red) and the ERGIC (green) in ZR75.1 cells and R-ZR75.1 cells treated with 2 ug/mL HT-DNA for 12 h. **D.** GSEA analysis of upregulated HALLMARK pathways in endocrine resistant breast cancer patients (GSE20181) and cell lines (GSE75971), NOM p-value <0.05.P-values were calculated by unpaired two-tailed Student’s T test, **p<0.01. NS, not significant.

Figure. S5.

**
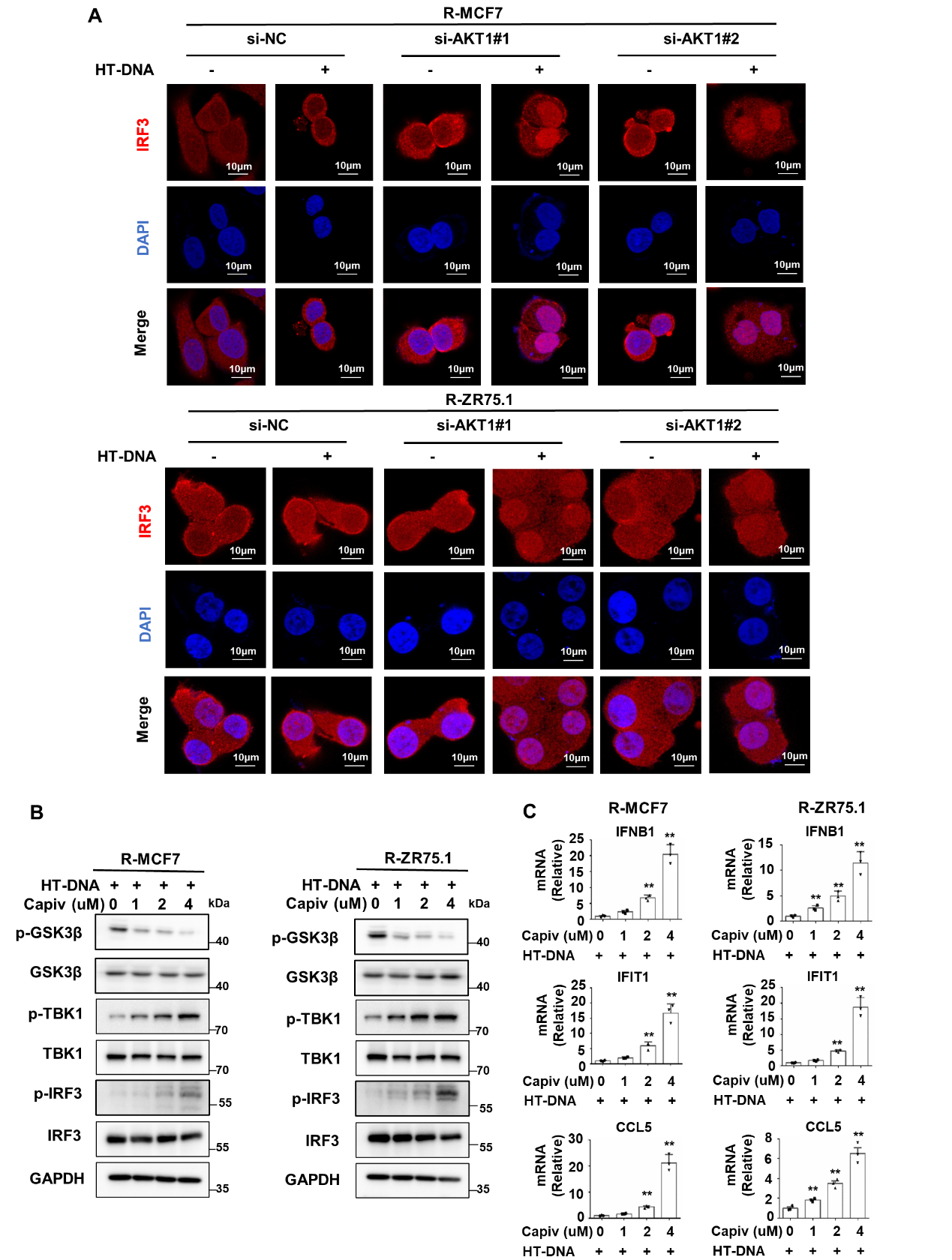
**

**Figure. S5. Targeting AKT1 reverses the activity of cGAS-STING pathway in endocrine-resistant breast cancer cells. A.** siAKT1 was used to interfere the expression of AKT1 in R-MCF7/R-ZR75.1 cells for 48 h, then cells were treated with 2 ug/mL HT-DNA for 12 h and harvested for immunofluorescence detection of IRF3 (red). **B.** R-MCF7/R-ZR75.1 cells were pretreated with capivasertib for 2 h, then cells were treated with 2 ug/mL HT-DNA for 12 h and harvested for western blot analysis of proteins in cGAS-STING pathway. **C.** R-MCF7/R-ZR75.1 cells were pretreated with capivasertib for 2 h, then cells were treated with 2 ug/mL HT-DNA for 12 h and harvested for for RT-qPCR analysis of IFNB1 mRNA, IFIT1 mRNA and CCL5 mRNA. P-values were calculated by unpaired two-tailed Student’s T test, **p<0.01. NS, not significant.

Figure. S6.


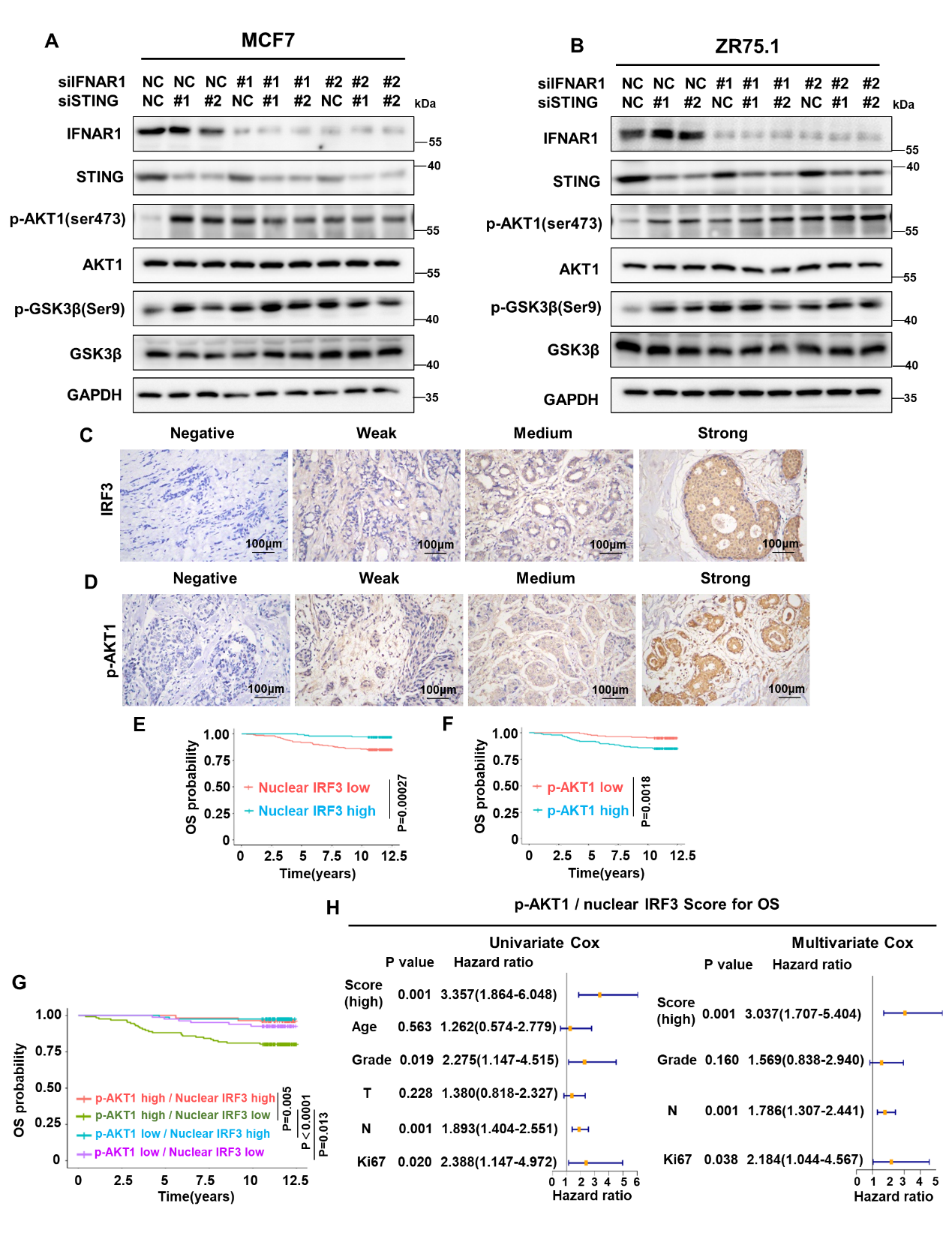


**Figure. S6. The prognostic significance of p-AKT1 and nuclear IRF3 in overall survival of ER+HER2- breast cancer.** Knockdown IFNAR1 and/or STING in MCF7**(A)** and ZR75.1**(B)** cells for 72 h, then cells were harvested for western blot analysis of proteins in PI3K-AKT pathway. **C.** The representative intensity images for each IHC score of nuclear IRF3 staining in ER+HER2- breast cancer tissues. **D.** The representative intensity images for each IHC score of p-AKT1 staining in ER+HER2- breast cancer tissues. **E.** Kaplan–Meier plots of the OS of patients, stratified by protein expression of nuclear IRF3. The p value was assessed using the log-rank test (two-sided). **F.** Kaplan–Meier plots of the OS of patients, stratified by protein expression of p-AKT1. The p value was assessed using the log-rank test (two-sided). **G.** Kaplan–Meier plots of the OS of patients, stratified by protein expression of both p-AKT1 and nuclear IRF3. The p value was assessed using the log-rank test and further corrected with the Benjamini–Hochberg method (two-sided). **H.** Univariate Cox regression analysis and multivariate Cox regression analysis regarding OS for ER+HER2- breast cancer patients.

**Figure. S7**


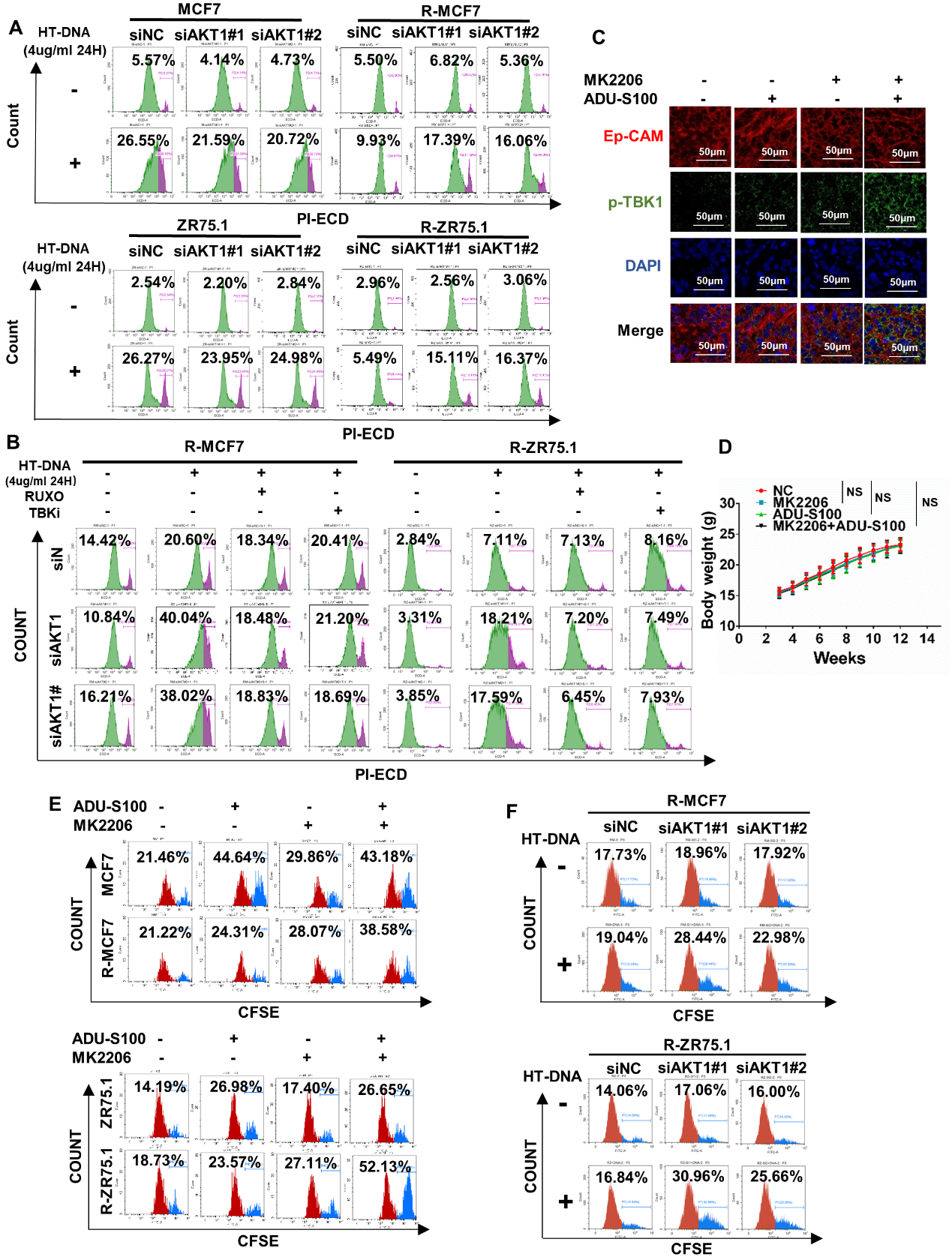


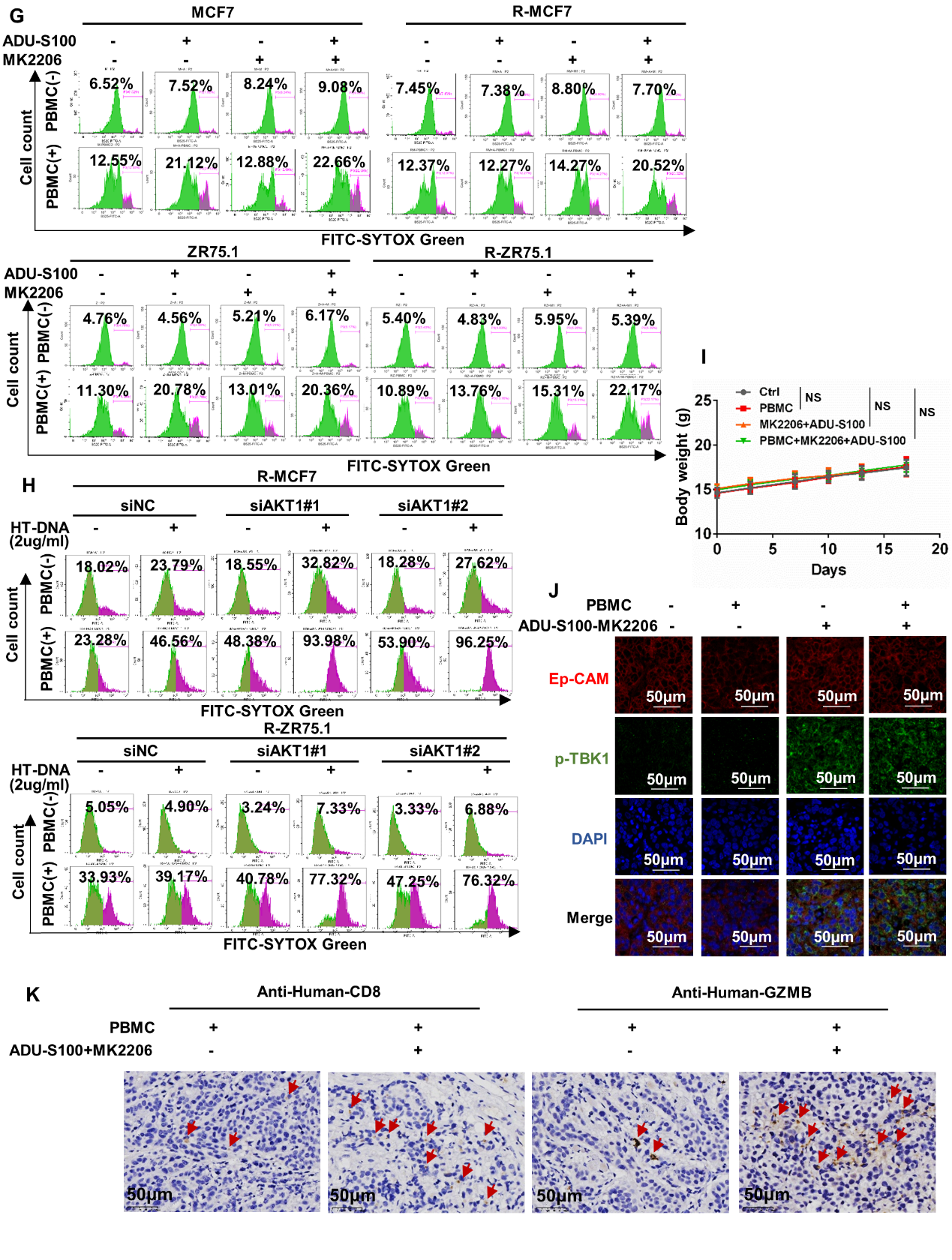


**Figure. S7. The combination of ADU-S100 and MK2206 enhances immune surveillance. A.** Flow cytometry measuring HT-DNA induced cell death of MCF7/ZR75.1 cells and R-MCF7/R-ZR75.1 cells after AKT1 knockdown. **B.** Knockdown AKT1 in R-MCF7/R-ZR75.1 cells for 48 h, then cells were pretreated with 0.5 uM Ruxolitinib or 1 uM GSK8612 for 2 h. After that cells were treated with 4 ug/ml HT-DNA for 24 h and harvested for Annexin V-FITC/PI staining assay. **C.** Immunofluorescence detection of p-TBK1 in tumor cells of tissues from nude mice. **D.** The body weight of nude mice during the experiment in different groups. **E.** Flow cytometry measuring the DCs that engulfing cancer cells pre-treated with ADU-S100 and MK2206. **F.** Knockdown AKT1 in R-MCF7/R-ZR75.1 cells for 48 h, then cells were pre-treated with 2 μg/mL HT-DNA for 6 h. After that cancer cells were dyed by CFSE and co-cultured with immature DC for 16 h and harvested for flow cytometry analysis of DC that engulfing the cancer cells. **G.** Flow cytometry measuring the cytotoxicity of PBMCs that killing the cancer cells pre-treated with ADU-S100 and MK2206. **H.** siAKT1 was used to interfere the expression of AKT1 in R-MCF7/R-ZR75.1 cells for 8 h, then cells were pre-treated with 2 μg/mL HT-DNA for 6 h. After that, cancer cells were co-cultured with PBMC (present with anti-CD3, anti-CD28 and IL-2) for 72 h and harvested for flow cytometry analysis of cell death rate in cancer cells. **I.** The body weight of humanized mice during the experiment in different groups. **J.** Immunofluorescence detection of p-TBK1 in tumor cells of tissues from NSG mice and humanized mice. **K.** The representative intensity images for IHC of CD8 staining and GZMB staining in endocrine-resistant breast cancer tissues from humanized mice. P-values were calculated by unpaired two-tailed Student’s T test, **p<0.01. NS, not significant.

**Table S1.**

**The results of kinase inhibitor library screen.**

| Kinase inhibitor | fold change to DMSO | Kinase inhibitor | fold change to DMSO | Kinase inhibitor | fold change to DMSO | Kinase inhibitor | fold change to DMSO |
| --- | --- | --- | --- | --- | --- | --- | --- |
| PHA-767491 | 0.076937 | Bakuchiol | 0.837509 | Milciclib (PHA-848125) | 1.043499 | Taselisib (GDC 0032) | 1.383331 |
| URMC-099 | 0.246322 | SU9516 | 0.837969 | LY2228820 | 1.045472 | Perifosine (KRX-0401) | 1.384074 |
| Masitinib (AB1010) | 0.25196 | PF-562271 | 0.839237 | Sodium Houttuyfonate | 1.047251 | Cabozantinib (XL184, BMS-907351) | 1.402193 |
| Fedratinib (SAR302503, TG101348) | 0.3153 | AG-1478 (Tyrphostin AG-1478) | 0.841399 | Palomid 529 (P529) | 1.04814 | BMS-265246 | 1.413576 |
| CUDC-907 | 0.328636 | OTS964 | 0.842508 | LDC000067 | 1.050342 | Miransertib (ARQ 092) HCl | 1.414389 |
| TG101209 | 0.361204 | BAY-61-3606 | 0.845181 | BLU9931 | 1.051157 | G-749 | 1.419333 |
| Tofacitinib (CP-690550,Tasocitinib) | 0.408633 | GW5074 | 0.845785 | Icotinib | 1.055554 | Akti-1/2 | 1.422742 |
| CEP-33779 | 0.411041 | Chloroquine Phosphate | 0.846985 | Vistusertib (AZD2014) | 1.055587 | GSK1059615 | 1.42774 |
| XL019 | 0.424398 | Chrysophanic Acid | 0.849483 | Lifirafenib (BGB-283) | 1.055955 | Saracatinib (AZD0530) | 1.43395 |
| S-Ruxolitinib (INCB018424) | 0.42692 | Bardoxolone Methyl | 0.849587 | AZD2858 | 1.056708 | Afuresertib (GSK2110183) | 1.437237 |
| WHI-P154 | 0.439129 | TAK-715 | 0.850814 | Go 6983 | 1.060592 | WZ4003 | 1.44454 |
| ERK5-IN-1 | 0.452589 | PND-1186 (VS-4718) | 0.851233 | Ensartinib (X-396) | 1.061483 | SF2523 | 1.44875 |
| NVP-BSK805 2HCl | 0.472069 | LDC4297 (LDC044297) | 0.853545 | Bay 11-7085 | 1.066071 | Pilaralisib (XL147) | 1.448877 |
| PP121 | 0.497431 | Psoralidin | 0.856744 | Daphnetin | 1.067362 | Crizotinib (PF-02341066) | 1.453824 |
| Ibrutinib (PCI-32765) | 0.515076 | AZD1208 | 0.857652 | S49076 | 1.070407 | LY3023414 | 1.455181 |
| Bisindolylmaleimide I (GF109203X) | 0.519833 | BAY 1217389 | 0.858768 | XL147 analogue | 1.073983 | Osimertinib (AZD9291) | 1.458149 |
| PRT-060318 2HCl | 0.535708 | PF-00562271 | 0.859161 | Neratinib (HKI-272) | 1.07487 | Thiazovivin | 1.459244 |
| XMD8-92 | 0.543545 | BMS-582949 | 0.860243 | TG003 | 1.076081 | 1-Azakenpaullone | 1.465195 |
| Peficitinib (ASP015K, JNJ-54781532) | 0.544015 | JNK-IN-8 | 0.861635 | AZ5104 | 1.079129 | PF-04691502 | 1.469228 |
| MLN2480 | 0.553925 | SGX-523 | 0.863207 | BI-78D3 | 1.079157 | TIC10 | 1.473269 |
| BGT226 (NVP-BGT226) | 0.556897 | NPS-1034 | 0.866382 | Pazopanib | 1.080021 | SAR405 | 1.493237 |
| Y-39983 HCl | 0.564158 | GSK2656157 | 0.866494 | NU7441 (KU-57788) | 1.081428 | TIC10 Analogue | 1.497422 |
| Cerdulatinib (PRT062070, PRT2070) | 0.572443 | MNS (3,4-Methylenedioxy-β-nitrostyrene, MDBN) | 0.867511 | Purvalanol A | 1.081566 | Dasatinib | 1.497642 |
| WH-4-023 | 0.575422 | PIK-294 | 0.87054 | GNF-5837 | 1.08159 | SC66 | 1.498903 |
| Fostamatinib (R788) | 0.580377 | WP1066 | 0.872944 | GNE-0877 | 1.082929 | PF-431396 | 1.514236 |
| Ro3280 | 0.582341 | Telatinib | 0.874797 | Losmapimod (GW856553X) | 1.085987 | Pelitinib (EKB-569) | 1.522036 |
| Oclacitinib | 0.585291 | TAK-285 | 0.876279 | Taxifolin (Dihydroquercetin) | 1.086322 | VX-745 | 1.529453 |
| K03861 | 0.591302 | KN-93 Phosphate | 0.876314 | Cabozantinib malate (XL184) | 1.087187 | Ipatasertib (GDC-0068) | 1.535802 |
| Pacritinib (SB1518) | 0.593373 | XMD8-87 | 0.878393 | ENMD-2076 | 1.091589 | AZD5363 | 1.537317 |
| Kenpaullone | 0.595816 | MLN0905 | 0.879178 | Buparlisib (BKM120, NVP-BKM120) | 1.091953 | PD98059 | 1.549335 |
| CNX-774 | 0.60403 | TDZD-8 | 0.88139 | FRAX597 | 1.092461 | A66 | 1.558055 |
| CCT137690 | 0.610923 | BAY 11-7082 | 0.882602 | TGR-1202 | 1.093762 | Flavopiridol (Alvocidib) | 1.560662 |
| PD173955 | 0.622329 | Anacardic Acid | 0.882735 | Axitinib | 1.09589 | BX-795 | 1.567986 |
| SKI II | 0.627522 | LY2603618 | 0.88665 | GNE-317 | 1.097433 | Brivanib (BMS-540215) | 1.578861 |
| AZD4547 | 0.62776 | HS-173 | 0.887621 | Torin 2 | 1.101261 | Vatalanib (PTK787) 2HCl | 1.58399 |
| THZ1 2HCl | 0.633058 | VX-702 | 0.888258 | FIIN-2 | 1.101775 | Refametinib (RDEA119, Bay 86-9766) | 1.602695 |
| MSC2530818 | 0.634032 | Amcasertib (BBI503) | 0.889477 | Afatinib (BIBW2992) Dimaleate | 1.110758 | SNS-032 (BMS-387032) | 1.611798 |
| KN-62 | 0.635772 | GNF-5 | 0.889635 | PRT062607 (P505-15, BIIB057) HCl | 1.110925 | Cediranib (AZD2171) | 1.615978 |
| AT7519 HCl | 0.636159 | Bafetinib (INNO-406) | 0.891463 | AZD1080 | 1.114353 | KU-55933 (ATM Kinase Inhibitor) | 1.61674 |
| UNC2250 | 0.636403 | Bosutinib (SKI-606) | 0.892428 | INK 128 (MLN0128) | 1.114801 | Rigosertib (ON-01910) | 1.62625 |
| P276-00 | 0.638518 | CGK 733 | 0.89257 | VX-11e | 1.115868 | PI-103 | 1.637996 |
| ONO-4059 (GS-4059) hydrochloride | 0.644258 | Picropodophyllin (PPP) | 0.892746 | WAY-600 | 1.118317 | JNJ-38877605 | 1.641932 |
| Anisomycin | 0.648304 | MK-8745 | 0.893017 | ZM 323881 HCl | 1.118575 | BMS-777607 | 1.643965 |
| LY2857785 | 0.652738 | GNE-9605 | 0.893662 | Voxtalisib (XL765, SAR245409) | 1.120168 | AR-A014418 | 1.649426 |
| MK-5108 (VX-689) | 0.657591 | HTH-01-015 | 0.894503 | HER2-Inhibitor-1 | 1.120418 | SB203580 | 1.64969 |
| GSK1070916 | 0.660772 | NSC 23766 | 0.89665 | Dabrafenib Mesylate | 1.121702 | PLX-4720 | 1.656822 |
| NH125 | 0.666118 | EHop-016 | 0.896652 | Dovitinib (TKI258) Lactate | 1.122892 | Genistein | 1.664827 |
| Tandutinib (MLN518) | 0.666164 | GSK2606414 | 0.898151 | TCS 359 | 1.12364 | IPI-549 | 1.665758 |
| NU6027 | 0.669766 | RKI-1447 | 0.898247 | WZ4002 | 1.126272 | Gefitinib (ZD1839) | 1.671567 |
| BMS-345541 | 0.67149 | SB239063 | 0.900315 | Carbazochrome | 1.129369 | LY294002 | 1.683494 |
| Dinaciclib (SCH727965) | 0.672793 | GSK2578215A | 0.901117 | CCT196969 | 1.130599 | Tie2 kinase inhibitor | 1.689218 |
| AZD5438 | 0.674151 | Capmatinib (INCB28060) | 0.90177 | Poziotinib (HM781-36B) | 1.131255 | BMS-794833 | 1.690437 |
| PP1 | 0.676341 | LTURM34 | 0.903516 | Alisertib (MLN8237) | 1.132712 | Enzastaurin (LY317615) | 1.697875 |
| Baricitinib (LY3009104, INCB028050) | 0.676369 | AZD3759 | 0.911277 | BFH772 | 1.13634 | Uprosertib (GSK2141795) | 1.707185 |
| Nintedanib (BIBF 1120) | 0.679375 | 10058-F4 | 0.916488 | Schisandrin B (Sch B) | 1.138704 | AZD1480 | 1.709839 |
| ML167 | 0.679801 | CNX-2006 | 0.916761 | AG-18 | 1.141138 | Ruxolitinib (INCB018424) | 1.710311 |
| Flavopiridol HCl | 0.679867 | Honokiol | 0.921132 | CHIR-98014 | 1.14286 | Linsitinib (OSI-906) | 1.710568 |
| Tanzisertib(CC-930) | 0.681618 | PF-3758309 | 0.924324 | (-)-Epigallocatechin Gallate | 1.145325 | AZD8330 | 1.714639 |
| Silmitasertib (CX-4945) | 0.689528 | ZINC00881524 (ROCK inhibitor) | 0.925437 | Pexmetinib (ARRY-614) | 1.148063 | MGCD-265 | 1.716225 |
| Go6976 | 0.690445 | CCT245737 | 0.925441 | DDR1-IN-1 | 1.148203 | BS-181 HCl | 1.716382 |
| RO9021 | 0.695283 | PF-543 | 0.927106 | SU6656 | 1.148245 | AZ 960 | 1.718788 |
| IC261 | 0.696349 | Irbinitinib (ARRY-380, ONT-380) | 0.928995 | CH5183284 (Debio-1347) | 1.148906 | BIX 02188 | 1.719224 |
| IKK-16 (IKK Inhibitor VII) | 0.697098 | R788 (Fostamatinib) Disodium | 0.929676 | RN486 | 1.149786 | AICAR (Acadesine) | 1.725259 |
| Dovitinib (TKI-258) Dilactic Acid? | 0.698026 | PIK-75 HCl | 0.932341 | Sennoside B | 1.14983 | GSK429286A | 1.743861 |
| Dabrafenib (GSK2118436) | 0.700638 | NMS-P937 (NMS1286937) | 0.934486 | Trapidil | 1.151971 | WZ8040 | 1.748413 |
| SC-514 | 0.704882 | Scopoletin | 0.935063 | LJI308 | 1.155538 | TWS119 | 1.74924 |
| Belizatinib (TSR-011) | 0.707658 | WYE-125132 (WYE-132) | 0.937119 | CH5132799 | 1.15556 | PHA-793887 | 1.768398 |
| Reversine | 0.708004 | GSK2982772 | 0.938438 | Tideglusib | 1.15821 | Ridaforolimus (Deforolimus, MK-8669) | 1.771393 |
| BMS-911543 | 0.709622 | PD168393 | 0.940561 | SKLB1002 | 1.159635 | Quizartinib (AC220) | 1.775881 |
| R547 | 0.710138 | Apatinib | 0.942807 | GW441756 | 1.161304 | Y-27632 2HCl | 1.778236 |
| Ruxolitinib Phosphate | 0.712753 | eFT-508 (eFT508) | 0.945984 | Vandetanib (ZD6474) | 1.162144 | PHA-665752 | 1.783357 |
| XMD16-5 | 0.713744 | A-769662 | 0.946664 | RO5126766 (CH5126766) | 1.166202 | Vemurafenib (PLX4032, RG7204) | 1.78764 |
| GSK269962A HCl | 0.715131 | DEL-22379 | 0.947128 | DASA-58 | 1.166839 | AZD7762 | 1.79267 |
| Raf265 derivative | 0.719418 | GDC-0994 | 0.947725 | ASP3026 | 1.168549 | TGX-221 | 1.797951 |
| CEP-32496 | 0.719922 | AMG-458 | 0.948203 | CAY10505 | 1.16884 | CP-724714 | 1.805936 |
| Myricetin | 0.723029 | AZ 628 | 0.948496 | PFK15 | 1.170262 | Sorafenib Tosylate | 1.806885 |
| Entrectinib (RXDX-101) | 0.724761 | AZ191 | 0.948857 | ETP-46464 | 1.171007 | Temsirolimus (CCI-779, NSC 683864) | 1.82265 |
| WNK463 | 0.725639 | ZM 39923 HCl | 0.953748 | SU5402 | 1.171681 | AMG319 | 1.824122 |
| Notoginsenoside R1 | 0.726083 | Lupeol | 0.954933 | Ceritinib (LDK378) | 1.176974 | SNS-314 Mesylate | 1.846053 |
| Entospletinib (GS-9973) | 0.72743 | AZ20 | 0.957784 | IM-12 | 1.181845 | SGI-1776 free base | 1.847784 |
| BI-D1870 | 0.727687 | Erdafitinib (JNJ-42756493) | 0.958019 | SSR128129E | 1.183691 | Nilotinib (AMN-107) | 1.854531 |
| Ripasudil (K-115) hydrochloride dihydrate | 0.72908 | GNF-2 | 0.958162 | Duvelisib (IPI-145, INK1197) | 1.18525 | Brivanib Alaninate (BMS-582664) | 1.868783 |
| DTP3 | 0.729174 | Golvatinib (E7050) | 0.96112 | Palbociclib (PD-0332991) HCl | 1.18545 | AT7519 | 1.872094 |
| PLX7904 | 0.731353 | 7,8-Dihydroxyflavone | 0.961776 | BIO | 1.187271 | Pimasertib (AS-703026) | 1.895464 |
| MK-2461 | 0.734082 | PF-06273340 | 0.961894 | GDC-0326 | 1.189531 | ZM 447439 | 1.908266 |
| STO-609 | 0.735499 | Pazopanib HCl (GW786034 HCl) | 0.962447 | Idelalisib (CAL-101, GS-1101) | 1.191562 | R406 (free base) | 1.910667 |
| FLLL32 | 0.73649 | AZD8835 | 0.963764 | ALK-IN-1 | 1.192965 | OSI-930 | 1.922331 |
| Amlexanox | 0.736527 | Lorlatinib?(PF-6463922) | 0.966564 | SU11274 | 1.196698 | PD0325901 | 1.92458 |
| Torkinib (PP242) | 0.736882 | AZD3463 | 0.96711 | Vacquinol-1 | 1.197176 | PF-4708671 | 1.927777 |
| Decernotinib (VX-509) | 0.737819 | PH-797804 | 0.969894 | Sunitinib | 1.200048 | Gandotinib (LY2784544) | 1.932749 |
| Piceatannol | 0.739909 | TAK-632 | 0.970085 | ANA-12 | 1.200164 | PF-573228 | 1.948041 |
| Larotrectinib (LOXO-101) sulfate | 0.749046 | PP2 | 0.971958 | VPS34 inhibitor 1 (Compound 19, PIK-III analogue) | 1.201082 | Linifanib (ABT-869) | 1.948747 |
| NU2058 | 0.751591 | PQ 401 | 0.973555 | Deguelin | 1.206174 | CC-223 | 1.949933 |
| Ro-3306 | 0.752026 | Dasatinib hydrochloride | 0.974257 | Shikonin | 1.206267 | AZD6482 | 1.95712 |
| TPCA-1 | 0.753547 | Pictilisib (GDC-0941) | 0.974446 | GSK2269557 | 1.206735 | Barasertib (AZD1152-HQPA) | 1.978391 |
| Dacomitinib (PF299804, PF299) | 0.75452 | BMS-536924 | 0.976995 | 7-Hydroxy-4-chromone | 1.206792 | SP600125 | 1.985671 |
| JNK Inhibitor IX | 0.756612 | OSI-027 | 0.977108 | SC1 | 1.207148 | PD184352 (CI-1040) | 1.991784 |
| A-674563 | 0.757452 | GNF-7 | 0.978101 | CCT128930 | 1.208419 | Fasudil (HA-1077) HCl | 2.003675 |
| Tepotinib (EMD 1214063) | 0.7577 | LY333531 HCl | 0.978106 | RXDX-106 (CEP-40783) | 1.211484 | R406 | 2.026942 |
| CX-6258 HCl | 0.760041 | Naquotinib(ASP8273) | 0.978135 | Alpelisib (BYL719) | 1.214146 | Roscovitine (Seliciclib,CYC202) | 2.028036 |
| abemaciclib (LY2835219) | 0.761001 | Rociletinib (CO-1686, AVL-301) | 0.980408 | ZM 306416 | 1.219749 | OSI-420 | 2.061476 |
| Chk2 Inhibitor II (BML-277) | 0.761074 | ONO-4059 analogue | 0.983661 | Semaxanib (SU5416) | 1.222158 | 3-Methyladenine (3-MA) | 2.087359 |
| OTS514 hydrochloride | 0.761297 | LRRK2-IN-1 | 0.983939 | CZC24832 | 1.229748 | BIX 02189 | 2.092519 |
| IPA-3 | 0.7637 | Tyrphostin AG 1296 | 0.984345 | SAR131675 | 1.23103 | Doramapimod (BIRB 796) | 2.100189 |
| ENMD-2076 L-(+)-Tartaric acid | 0.76427 | Encorafenib (LGX818) | 0.984776 | Sunitinib Malate | 1.23242 | GSK461364 | 2.106989 |
| BI-847325 | 0.764604 | GSK'872 (GSK2399872A) | 0.986117 | Hesperadin | 1.232671 | NVP-AEW541 | 2.134846 |
| PF-477736 | 0.766101 | Merestinib (LY2801653) | 0.986668 | Tozasertib (VX-680, MK-0457) | 1.235258 | Everolimus (RAD001) | 2.155741 |
| Cobimetinib (GDC-0973, RG7420) | 0.76641 | Skepinone-L | 0.98748 | AZD8186 | 1.236917 | AST-1306 | 2.15872 |
| Forsythin | 0.768735 | AS-604850 | 0.987881 | Miltefosine | 1.239688 | Sapitinib (AZD8931) | 2.162171 |
| Senexin A | 0.76936 | AT9283 | 0.988503 | NT157 | 1.24079 | AG-1024 | 2.171127 |
| SB590885 | 0.77145 | KD025 (SLx-2119) | 0.988806 | Apatinib | 1.242088 | KU-0063794 | 2.174679 |
| UNC2025 | 0.771861 | GSK2256098 | 0.990451 | GDC-0084 | 1.242233 | Lapatinib | 2.218901 |
| Verbascoside | 0.773255 | JNJ-7706621 | 0.992569 | KW-2449 | 1.245248 | U0126-EtOH | 2.219182 |
| PF-06447475 | 0.773922 | BLU-554 (BLU554) | 0.993253 | LJH685 | 1.248143 | PIK-93 | 2.221452 |
| Defactinib (VS-6063, PF-04554878) | 0.775947 | Pyridoxine | 0.99371 | GSK1838705A | 1.249173 | PD318088 | 2.225751 |
| GDC-0623 | 0.776218 | Asiatic Acid | 0.993756 | Imatinib (STI571) | 1.256388 | RAF265 (CHIR-265) | 2.235536 |
| Pseudolaric Acid B | 0.776409 | NU7026 | 0.993867 | Zotarolimus(ABT-578) | 1.257205 | Motesanib Diphosphate (AMG-706) | 2.253605 |
| TAK-901 | 0.778191 | Alectinib hydrochloride | 0.995862 | CYC116 | 1.258912 | Afatinib (BIBW2992) | 2.259281 |
| LDC1267 | 0.779096 | kira6 | 0.996284 | Serabelisib (INK-1117,MLN-1117,TAK-117) | 1.265304 | Lenvatinib (E7080) | 2.292679 |
| Tofacitinib (CP-690550) Citrate | 0.77931 | GZD824 Dimesylate | 0.996503 | BIO-acetoxime | 1.265408 | KU-60019 | 2.328011 |
| AMG-900 | 0.780641 | CL-387785 (EKI-785) | 0.997788 | TPX-0005 | 1.272084 | Ponatinib (AP24534) | 2.348493 |
| GSK180736A (GSK180736) | 0.786648 | CGP 57380 | 0.999283 | LFM-A13 | 1.274793 | NVP-BHG712 | 2.360777 |
| BAW2881 (NVP-BAW2881) | 0.78801 | Tyrphostin AG 879 | 1.000437 | PHA-680632 | 1.276283 | Foretinib (GSK1363089) | 2.380971 |
| AZD6738 | 0.789848 | Dorsomorphin 2HCl | 1.000769 | PIK-III | 1.281497 | AZD8055 | 2.391894 |
| FRAX486 | 0.794101 | ID-8 | 1.002761 | Dovitinib (TKI-258, CHIR-258) | 1.282031 | SB216763 | 2.394226 |
| GW2580 | 0.795873 | Bikinin | 1.003363 | LY2090314 | 1.2821 | HMN-214 | 2.40377 |
| AMG 337 | 0.796546 | SL-327 | 1.003661 | SB415286 | 1.283119 | TG100-115 | 2.415626 |
| TAE226 (NVP-TAE226) | 0.797702 | Obacunone | 1.004644 | Binimetinib (MEK162, ARRY-162, ARRY-438162) | 1.288299 | GDC-0879 | 2.43399 |
| Filgotinib (GLPG0634) | 0.799041 | Mubritinib (TAK 165) | 1.007132 | GSK2334470 | 1.288374 | GSK1904529A | 2.474216 |
| Radotinib | 0.799505 | GSK621 | 1.007767 | Butein | 1.288786 | PD173074 | 2.501664 |
| SBE 13 HCl | 0.799698 | Olmutinib (HM61713, BI 1482694) | 1.009258 | AT13148 | 1.289746 | CUDC-101 | 2.5437 |
| Phenformin HCl | 0.802151 | GSK650394 | 1.010135 | PF-04217903 | 1.291456 | Amuvatinib (MP-470) | 2.547585 |
| Fingolimod (FTY720) HCl | 0.803385 | VE-821 | 1.01116 | CZ415 | 1.300166 | PIK-293 | 2.555986 |
| CGI1746 | 0.803634 | Erlotinib | 1.014753 | BLZ945 | 1.30035 | WYE-354 | 2.584583 |
| Varlitinib | 0.803696 | GNE-7915 | 1.015134 | Apitolisib (GDC-0980, RG7422) | 1.302104 | Tivozanib (AV-951) | 2.599553 |
| Quercetin | 0.805254 | CFI-400945 | 1.016785 | Nintedanib Ethanesulfonate Salt | 1.303705 | CHIR-99021 (CT99021) | 2.605958 |
| Tyrphostin 9 | 0.806047 | Hydroxyfasudil (HA-1100) HCl | 1.018874 | Adenosine 5'-monophosphate monohydrate | 1.306528 | CP-673451 | 2.639874 |
| Berbamine (dihydrochloride) | 0.807299 | UNC2881 | 1.020756 | Aurora A Inhibitor I | 1.308058 | H 89 2HCl | 2.670589 |
| D 4476 | 0.807551 | CC-115 | 1.021012 | VPS34-IN1 | 1.309035 | Orantinib (TSU-68, SU6668) | 2.718306 |
| IMD 0354 | 0.807586 | Gilteritinib (ASP2215) | 1.023617 | AG-490 (Tyrphostin B42) | 1.324615 | KRN 633 | 2.733043 |
| PD-166866 (PD166866) | 0.808165 | VS-5584 (SB2343) | 1.025319 | Sorafenib | 1.325196 | ZSTK474 | 2.805681 |
| AS-252424 | 0.808216 | EAI045 | 1.025853 | LY2874455 | 1.325415 | AEE788 (NVP-AEE788) | 2.843182 |
| ML-7 HCl | 0.813298 | Glabridin | 1.02709 | CHIR-99021 (CT99021) HCl | 1.327042 | Regorafenib (BAY 73-4506) | 2.866183 |
| NVP-BVU972 | 0.815043 | Indirubin | 1.027662 | WZ3146 | 1.327097 | OSU-03012 (AR-12) | 2.930673 |
| Pexidartinib (PLX3397) | 0.815111 | AD80 | 1.028004 | Imatinib Mesylate (STI571) | 1.334009 | Ki8751 | 3.05668 |
| Autophinib | 0.815381 | Necrosulfonamide | 1.028599 | PF-4989216 | 1.334099 | YM201636 | 3.134048 |
| LY2584702 Tosylate | 0.816369 | KX2-391 | 1.028822 | MLN8054 | 1.33472 | AT7867 | 3.432869 |
| Momelotinib (CYT387) | 0.817566 | Sotrastaurin | 1.029706 | GSK2292767 | 1.339964 | Voxtalisib | 3.497455 |
| Nazartinib (EGF816, NVS-816) | 0.818021 | TAK-733 | 1.030873 | Omipalisib (GSK2126458, GSK458) | 1.34663 | Triciribine | 3.622031 |
| CC-292 (AVL-292) | 0.822276 | Pamapimod (R-1503, Ro4402257) | 1.03131 | Sitravatinib (MGCD516) | 1.35295 | BX-912 | 3.715252 |
| ETC-1002 | 0.825176 | VE-822 | 1.031347 | AZD2932 | 1.366908 | PHT-427 | 3.737793 |
| Degrasyn (WP1130) | 0.829386 | Crenolanib (CP-868596) | 1.031351 | SB202190 (FHPI) | 1.367051 | MK-2206 | 3.993837 |
| SGI-7079 | 0.830051 | NCB-0846 | 1.032474 | GDC-0349 | 1.374184 | GSK690693 | 5.132645 |
| LDN-214117 | 0.831421 | PRN1371 | 1.036505 | Lapatinib (GW-572016) Ditosylate | 1.379858 |  |  |
| SAR-020106 | 0.836121 | GSK2636771 | 1.040035 | PI-3065 | 1.381324 |  |  |
| Acalabrutinib (ACP-196) | 0.837423 | ZM 336372 | 1.042034 | XL388 | 1.381598 |  |  |

Table S2.

**The clinical characteristics of 4 patients for spatial transcriptomic analyses.**

| **Patients** | **Patient 1 with endocrine-sensitive BC** | **Patient 2 with endocrine-sensitive BC** | **Patient 1 with endocrine- resistant BC** | **Patient 2 with endocrine-resistant BC** |
| --- | --- | --- | --- | --- |
| **sample ID** | BC860 | BC1670 | BC1780 | BC2558 |
| **Age** | 59 | 53 | 61 | 44 |
| **Gender** | female | female | female | female |
| **Menstruation** | Post-menopause | Pre-menopause | Post-menopause | Pre-menopause |
| **Pathology** | invasive ductal carcinoma | invasive ductal carcinoma | invasive ductal carcinoma | invasive ductal carcinoma |
| **ER+ (%)** | 70% | 90% | 80% | 90% |
| **ER status** | positive | positive | positive | positive |
| **HER2 status** | negative | negative | negative | negative |
| **Drug for ET** | letrozole | Tamoxifen- letrozole | anastrozole | tamoxifen |
| **Duration of ET (years)** | 7 | 5 | 5.5 | 2 (Dead at 2 years) |
| **Relapse** | No | No | Yes | Yes |
| **Time from beginning of ET to relapse (years)** | NA | NA | 2.5 | 0.5 |
| **Abbreviation:** BC, breast cancer; ER, estrogen receptor; HER2, Human epidermal growth factor receptor 2; ET, endocrine therapy. | | | | |

Table S3.

**Antibodies and Resource.**

| Antibodies | Source | Identifier | Dilution |
| --- | --- | --- | --- |
| HER2 | Santa Cruz | sc-33684 | 1:500 |
| cGAS | Proteintech | 26416-1-AP-100UL | 1:1000 |
| TMEM173/STING | Proteintech | 19851-1-AP-100UL | 1:1000 |
| IRF3 | Proteintech | 11312-1-AP-100UL | 1:1000 |
| TBK1 | Cell signaling technology | 38066S | 1:1000 |
| [AKT1 (B-1）](javascript:void(0)) | Santa Cruz | sc-5298 | 1:500 |
| STAT1 | Proteintech | 66545-1-IG-50UL | 1:1000 |
| Phospho-TBK1 (Ser172) | Cell signaling technology | 5483S | 1:1000 |
| Phospho-STING (Ser366) | Cell signaling technology | 19781S | 1:1000 |
| Phospho-IRF-3 (Ser396) | Cell signaling technology | 29047S | 1:1000 |
| Phospho-Stat1 (Ser727) | Cell signaling technology | 9177S | 1:1000 |
| Phospho-IRF-3 (Ser386) | Cell signaling technology | 37829S | 1:1000 |
| Phospho-AKT (Ser473) | Cell signaling technology | 4060S | 1:1000 |
| GAPDH | Proteintech | 60004-1-IG-100UL | 1:5000 |
| α-tubulin | Beyotime | AT819 | 1:5000 |
| DYKDDDDK Tag (FALG) | Cell signaling technology | 14793S | 1:1000 |
| HA-Tag | Cell signaling technology | 3724S | 1:1000 |
| Myc-Tag | Cell signaling technology | 2276S | 1:1000 |
| GFP-Tag | Cell signaling technology | 2037S | 1:1000 |
| CD8a | Proteintech | 66868-1-IG | 1:300 |
| GZMB | Proteintech | 13588-1-AP | 1:300 |

**Methods**

**Plasmids**

Human full-length STING (fused C-terminal FLAG tag), TBK1 (fused C-terminal MYC tag) and IRF3 (fused C-terminal GFP tag) were subcloned into pcDNA3.1 vector (Invitrogen) for transient transfection. Using the human pcDNA3.1-TBK1-MYC plasmid as a template, truncation mutant plasmids pcDNA3.1-TBK1-1-299-MYC, pcDNA3.1-TBK1-1-382-MYC and pcDNA3.1-TBK1-299-729-MYC were developed. pcDNA3.1-HA-AKT1, pcDNA3.1-HA-DN-AKT1, pcDNA3.1-HA-myr-AKT1 were previously generated in our laboratory. Using the human pcDNA3.1-HA-AKT1 plasmid as a template, truncation mutant AKT1-1-148, AKT1-1-412 and AKT1-149-480 were subcloned into pSin-SFB vector. pRL-5×ISRE -Luc plasmid was a gift from Xu’s laboratory. All plasmids were generated by using the ClonExpress II One Step Cloning Kit (C112-01) and ClonExpress Multis One Step Cloning Kit (C113-01) from Vazyme. All plasmids were verified by DNA sequencing. and all mutations were verified by DNA sequencing.

**Spatial transcriptomics using Stereo-seq**

For spatial transcriptomics experiments, Stereo-seq chips were used. Four samples of primary breast cancer were embedded in Tissue-Tek OCT (Sakura, 4583) and stored at -80℃. Fluorescent chip was used to find the optimal permeabilization time was 12 min. Tissue was sectioned at 10μm and adhered to the gene expression chip, whereas the adjacent sections were used for H&E staining. The chip was placed onto a warming plate at 37 °C for 3 min for drying and immersed in precooled methanol at −20 °C for 30 min for fixation. For permeabilization, the chip was incubated with 100 μl 0.1% pepsin (Sigma-Aldrich, P7000) at 37 °C for 12 min and washed with 0.1× SSC buffer (Thermo Fisher Scientific, AM9770) containing 0.05 U μl^−1^ RNase inhibitor. Released RNA from tissues was captured by DNB and reverse-transcribed for 90 min at 42 °C using 100 μl SuperScript II mix (10 U μl^−1^ reverse transcriptase, 1 mM dNTPs, 1 M betaine solution PCR reagent, 7.5 mM MgCl2, 5 mM DTT, 2 U μl^−1^ RNase inhibitor, 2.5 μM stereo-seq template switch oligo, and 1× first-strand buffer). Tissue on the chip was then removed by incubating with tissue removal buffer (10 mM Tris-HCl, 25 mM EDTA, 100 mM NaCl, 0.5% SDS) at 37°C for 30 min and the chip was washed using 0.1× SSC buffer twice. The chip was incubated with 400 μl cDNA release buffer at 55 °C for 4 h. The cDNA was then purified using AMPure XP DNA Clean Beads (Vazyme, N411-03) and amplified using KAPA HiFi Hotstart Ready Mix (Roche, KK2602) and cDNA primers. PCR product was fragmented to generate the cDNA sequencing library. The cDNA library was loaded onto a DNBSEQ-T10 sequencer (MGI) for sequencing (pair-end, read 1 of 50 bp and read 2 of 100 bp). For celltype annotation, SPOTlight v0.1.7 package was applied to deconvolute celltype compositions on Bin200 spots(Bin200 resolution, 100 μm diameter). First, spatial transcriptomics expression matrix was transformed into a Seurat object and we discarded low-quality spots with a high percentage of [mitochondrial genes](https://www.sciencedirect.com/topics/biochemistry-genetics-and-molecular-biology/mitochondrial-gene) (>20%) and a low gene number(<500). Then, a published breast cancer scRNA dataset was used as the reference input to SPOTlight. Thus, we acquired the cell proportion of each spot. Spots with more than 15% of cancer epithelial cells were defined as tumor region, which was confirmed by a pathologist inspecting the adjacent section H&E staining image. For scoring and statistical analysis, ssGSEA scoring of the pathway gene sets was performed on tumor region using GSVA package. Statistical test of scores between two groups was using Wilcoxon rank-sum tests. Pearson correlation coefficient was calculated between ssGSEA scores and SPOTlight deconvoluted celltype proportions. The spatial plot of score was using SpatialFeaturePlot function in Seurat package.

**siRNA transfection**

The cells were seeded into six-well plates the day before transfection. Transfection of siRNA was performed with lipofectamine RNAimax (Invitrogen) according to the manufacturer’s instruction. Oligonucleotide sequence of siRNAs was as following: siAKT1#1: 5′-GAGUUUGAGUACCUGAAGCUG-3′; siAKT1#2: 5′-AAGGAGGGUUGGCUGCACAAA-3′; siSTING#1: 5′-CCAACAUUCGCUUCCUGGAUA-3′; siSTING#2: 5′-GCAGAGCUAUUUCCUUCCACA-3′; siIFNAR1#1: 5′-GCCAAGAUUCAGGAAAUUAUU-3′; siIFNAR1#2: 5′-CCUUAGUGAUUCAUUCCAUAU-3′

**Quantitative real-time PCR**

Total RNA was extracted using RNA Purification Kit (B0004D, EZBioscience), and reverse transcription was performed using a HiScript II Q RT Kit (R223-01, Vazyme Biotech). Real-time PCR was performed using ChamQ SYBR qPCR Green Master Mix (Q311-03, Vazyme Biotech). and run with a Light Cycler 480 instrument (Roche Diagnostics). The relative amount of target gene mRNA was normalized to GAPDH. All qPCR reactions were done in triplicate.

| Gene | Sequence |
| --- | --- |
| human IFNB1 sense | 5′-GTCAGAGTGGAAATCCTAAG-3′ |
| human IFNB1 antisense | 5′-TATGCAGTACATTAGCCATC-3′ |
| human ISG15 sense | 5′-GAACTCATCTTTGCCAGTA-3′ |
| human ISG15 antisense | 5′-ATCTTCTGGGTGATCTGC-3′ |
| human IFIT1 sense | 5′-TACAGCAACCATGAGTACAA-3′ |
| human IFIT1 antisense | 5′-TCACATAGGCTAGTAGGTTG-3′ |
| human CXCL10 sense | 5′-TACCTGCATCAGCATTAGTA-3′ |
| human CXCL10 antisense | 5′-TGTAGCAATGATCTCAACAC-3′ |
| human CCL5 sense | 5′-AGCAGTCGTCTTTGTCAC-3′ |
| human CCL5 antisense | 5′-TAGCTCATCTCCAAAGAGTT-3′ |
| human GAPDH sense | 5′-GATTCCACCCATGGCAAATTC-3′ |
| human GAPDH antisense | 5′-CTTCTCCATGGTGGTGAAGAC-3′ |

**Immunoblot and immunoprecipitation**

The process of immunoblot and immunoprecipitation was described previously. Briefly, cells were harvested and lysed in RIPA buffer (Cat#9806s, Cell Signaling Technology) containing 1 mM phenylmethanesulfonyl fluoride. Total proteins were separated by SDS-PAGE and transferred to PVDF membrane. For co-immunoprecipitation, cells were harvested and lysed in Pierce IP Lysis Buffer (Thermo Scientific) supplemented with Complete Protease Inhibitor Cocktail (Roche). Whole-cell lysates were used for immunoprecipitation with the indicated antibodies. Generally, 1–2 μg of commercial antibody was added to 1 mg of total protein, and the mixture was incubated at 4 °C for overnight. After adding protein A/G agarose beads, the incubation was continued for 1 h. Antibodies for immunoblot were used at a dilution of 1:500–1:1000. Antibodies used in immunoblot and immunoprecipitation were listed Supplementary Table 1.

**Dual-luciferase reporter assay**

Cells were transfected with the 5×ISRE reporters bearing an ORF coding for the Firefly luciferase along with the pRL-Luc with the Renilla luciferase ORF as the internal control for transfection. At 12 h post transfection, the cells were treated with the indicated compounds. Then cells were lysed in a passive lysis buffer (Promega). Luciferase activity was measured using the Dual-Glo Luciferase Assay system (Promega) according to the manufacturer’s guidelines. Firefly luciferase activity was normalized to renilla luciferase to control for cell number and transfection efficiency.

**Immunofluorescence**

The process of Immunofluorescence was described previously. Briefly, cells were plated on coverslips and fixed in 4% (vol/vol) paraformaldehyde for 10 min and permeabilized with 0.1% triton-X-100 and block in 4% BSA for 45 mins. Then cells were incubated with corresponding primary antibodies overnight at 4℃, washed three times for 5 min each time, incubated in secondary antibodies for 1 h at room temperature. Secondary antibody washed and stained by DAPI and mounted on glass slides with ProLong™ Diamond Antifade Mountant (Invitrogen, P36970).

**Cell proliferation**

For MTT assay, the cells were seeded in 96-well plate (Falcon). Cell viability was determined by MTT. Briefly, MTT was added to each well for another 4 h at 37 °C. After that, MTT solution was removed and replaced with 150 μl DMSO. Absorbance values with a test wavelength of 570 nm and a reference wavelength of 650 nm was read by SpectraMax Plus 384 (MD). For colony formation assay, cells were seeded in a six-well plate and cultured for 1-2 week in indicated medium. Colonies were fixed and dyed with 0.1% crystal violet, and the number of colonies were counted.

**cGAMP ELISA**

cGAMP ELISA (Cayman Chemical) was performed on cell lysates according to the manufacturer’s instructions. Briefly, the same number of cells were taken from each sample for lysis. Then the concentration of cGAMP in the lysate was measured with cGAMP ELISA kit. Three technical replicates were used per biological replicate.

**Primary cells culture and stimulation**

Human peripheral blood mononuclear cells (PBMCs) were isolated from peripheral blood by Ficoll gradient centrifugation (17144002, GE Healthcare) and subjected to red blood cells lysis using RBC Lysis Buffer (00-4333-57, Thermo Fisher). Human DC cells were expanded by culturing PBMCs. PBMC was cultured in AIM-V medium (A3021002, Thermo Fisher) for 2 hours, the suspended cells in the medium were removed, and AIM-V medium supplemented with 1000 IU /ml GM-CSF and 500 IU/ml IL-4 were added to the adherent cells for induction of DC cells. After three days of induction, 500 IU/ml GM-CSF and 250 IU/ml IL-4 were added into the medium, and the semi-mature DC cells were obtained after continued induction for three days.

**Cytotoxicity assays in vitro**

Isolated PBMCs were cultured in AIM-V medium supplemented with Interleukin-2 (Cat# 200-02, Peprotech) in the 24-well-plate pre-coated with anti-CD3 (Cat#300313, BioLegend) and anti-CD28 (Cat# 302913, BioLegend). Then, the prepared tumor cells and PBMCs were co-cultured in the 24-well-plate at a ratio of 1:10 in triplicates. After co-cultured for 72 h, the cells were fixed, then stained with SYTOX Green. Anti-APC-CD45 was used to label CD45- tumor cells at the same time.

**DC maturation and phagocytosis assay**

For dendritic maturation assay, prepared tumor cells were cocultured with the semi-mature DCs for 24 hours at a ratio of 1:1. Then the mixture cells were subjected to flow cytometry to detect markers of mature DCs (CD11c, MHC-II). For DC phagocytosis assay, prepared tumor cells were stained with CFSE (Invitrogen) at 37°C for 15 minutes, and then cocultured with the semi-mature DCs for 16 hours at a ratio of 1:1. These mixture cells were subjected to flow cytometry to detect CD11c+ CFSE+ cells.

**Animal treatment protocol**

6- to 8-week-old female BALB/c nude mice were obtained from Gempharmatech-GD Company. All procedures involving nude mice and experimental protocols were approved by Institutional Animal Care and Use Committee (IACUC) of Sun Yat-sen University Cancer Center. All tumor cells were mixed with matrigel (1:1) and injected into the mammary fat fad of nude mice. To validate the combined effect of targeting AKT1 and STING agonist in vivo, 5x10^6^ R-MCF7 cells were injected into the mammary fat fad of each nude mouse. After xenograft tumors were palpable, the animals were randomly divided into four treatment groups: placebo+PBS, MK2206+PBS, placebo+ADU-S100, and MK2206+ADU-S100. The MK2206 was given by oral gavage at dose of 100mg/kg for each nude mouse. ADU-S100 was given by intratumor injection at dose of 5μg for each nude mouse. The frequency of treatment was twice a week. Tumor volumes and body weight of mice were observed. Volumes were calculated by the formula: 0.5 × A × B^2^ in millimeters, where A is the length and B is the width. After nude mice were killed, the tumor tissues were excised and weighed.

**Analyses of GEO and TCGA data**

The mRNA expression data and clinical data of TCGA breast cancer samples were downloaded from the Cancer Genome Atlas [TCGA: https://portal.gdc.cancer.gov/]. The mRNA expression data and clinical data of breast cancer patients receiving neoadjuvant endocrine therapy were downloaded from the Gene Expression Omnibus database [GEO: GSE20181]. The mRNA expression data of Long term estrogen deprived breast cancer cell lines were downloaded from the Gene Expression Omnibus database [GEO: GSE75971]. R software (version 4.0.3) was used for bioinformatics analysis. CIBERSORT deconvolution algorithm was used to analyze the infiltration of 22 types of immune cells in breast cancer tissues of patients in GSE20181. GSEA enrichment analysis was used to analyze gene sets that differed in enrichment between endocrine sensitive breast cancer and endocrine-resistant breast cancer. The ssGSEA algorithm was used to calculate the enrichment score of innate immune-related gene set for each luminal A breast cancer patient in the TCGA dataset. The package “Limma” was used to analyze differential genes between parental cells and endocrine-resistant cells.
